# Transcription-Coupled Nucleotide Excision Repair: A Faster Solution or the Only Option?

**DOI:** 10.3390/biom15071026

**Published:** 2025-07-16

**Authors:** Andriy Khobta, Leen Sarmini

**Affiliations:** Institute of Nutritional Sciences, Friedrich Schiller University Jena, 07743 Jena, Germany; leen.sarmini@uni-jena.de

**Keywords:** transcription-coupled nucleotide excision repair (TC-NER), DNA damage, endogenously generated DNA damage, DNA repair, DNA damage sensing, illudin S, acylfulvenes, cyclopurines, aldehyde genotoxicity, Cockayne syndrome (CS)

## Abstract

A branch of the nucleotide excision repair (NER) pathway, transcription-coupled repair (TCR or TC-NER) specifically operates on the template DNA strand of actively transcribed genes. Initiated by stalling of elongating RNA polymerase complexes at damaged sites, TC-NER has historically been viewed as “accelerated repair”, arguably necessary for the maintenance of vital transcription function. Conversely, the conventional “global genome” (GG-NER) mechanism, operating throughout the genome, is usually regarded as a much slower process, even though it has long been found that differences in repair kinetics between transcribed DNA and the rest of the genome are not manifested for all structural types of DNA damage. Considering that damage detection is the rate-limiting step of overall repair reactions in most cases and that the mechanisms of the initial recognition of modified DNA structure are fundamentally different between TC-NER and GG-NER, it is suggestive to attribute the observed kinetic differences to different damage spectra recognized by the two pathways. This review summarizes current knowledge on the differential requirements of TC-NER and GG-NER towards specific damage types, based on their structural rather than spatial characteristics, and highlights some common features of DNA modifications repaired preferentially or exclusively by TC-NER, while evading other repair mechanisms.

## 1. Introduction

Genome protection from accumulating mutations demands efficient mechanisms for the removal of damage that inevitably arises in DNA by action of radiation, environmental or dietary mutagens, reactive metabolites, as well as by spontaneous chemical decay of deoxyribonucleotides [1]. Base excision repair (BER), nucleotide excision repair (NER), and mismatch repair (MMR) constitute the first line of defense by removing modifications affecting only one DNA strand, whereby the non-damaged DNA strand serves as a template for faithful DNA re-synthesis [2,3,4,5]. Whereas BER and MMR enzymes have evolved to specifically recognize a limited set of the most commonly occurring DNA modifications or erroneous base pairs, NER is peculiar in its capacity to detect a formidable diversity of structurally different damage types—from endogenously arising modifications resistant to BER to a broad spectrum of DNA adducts generated by a variety of electrophilic substances or radiation sources, including UV light from the sun [6,7,8,9,10]. Accordingly, genetic defects in NER components, and, probably, oversaturation of repair capacities of heavily damaged cells, result in persistence of unrepaired damage, accumulation of mutations, and cellular stress responses, leading in the long term to cancer, aging, and degenerative diseases [7,11,12,13,14,15]. Thus, understanding the prerequisites and limits of versatile damage recognition by NER is immensely important.

A specific branch of the NER pathway termed “transcription-coupled repair” (TC-NER) is peculiar in several respects. Homozygous loss-of-function mutations in the key TC-NER genes *CSA* and *CSB* are causally associated with Cockayne syndrome, a severe hereditary disease with features of accelerated aging and neurodegeneration [16,17]. However, the exact relationship between the TC-NER defect and the Cockayne syndrome pathogenesis is not unequivocally understood, despite the intense research. Initially discovered as an accelerated removal of cyclobutane pyrimidine dimers (CPDs)—a subtype of DNA damage induced by UV radiation—from the template strand of actively transcribed genes, TC-NER has since then been regarded primarily as a mechanism for the rapid recovery of essential transcription, preceding the damage removal by the by the purportedly slower "global genome repair" pathway (GG-NER) [18]. Differently from all other repair pathways, TC-NER does not rely on a specialized DNA repair component for the damage detection but rather on the intrinsic structural properties of the RNA polymerase (RNAP) enzyme. This implies different structural determinants for the recognition of DNA damage compared to the universal GG-NER pathway, which relies on the specific damage detection proteins, as discussed in subsequent sections. Recent research has brought to light several classes of TC-NER substrates that are poorly processed by GG-NER, if at all. Instead of a delayed repair, this would imply complete inability of cells to remove such DNA lesions beyond the sequences directly read by transcribing RNAP (in eukaryotes, specifically RNAP II).

The scope of this review is to provide a survey of structural types of DNA lesions preferentially or exclusively processed by TC-NER in mammalian cells. We briefly summarize recent progress in the understanding of the peculiarities of the multistage damage recognition mechanisms of GG-NER and TC-NER. We further provide an overview of methods available for the investigation of TC-NER and of the most distinctive types of DNA lesions identified as TC-NER substrates while evading other repair mechanisms. Finally, we outline some common structural features characteristic of obligate TC-NER substrates characterized thus far.

## 2. Multistep Damage Recognition in NER

The universal NER damage sensing mechanism relies on the xeroderma pigmentosum group C-complementing (XPC) protein. In complex with RAD23A/B and CETN2, it probes DNA helix propensity to melt locally, thus allowing a stable XPC binding to the undamaged DNA strand opposite the modification [19,20,21,22]. This mechanism operates throughout the genome and hence is often referred to as “global genome NER” (GG-NER or GGR). In some cases of DNA modifications, such as UV-induced CPD, the degree of helix destabilization necessary for an efficient XPC binding is achieved with the assistance of the specific tetrameric UV-DDB complex, composed of the damage binding protein DDB2 and the DDB1-CUL4A-RBX1 (CRL4) ubiquitin ligase [23,24,25]. However, not all damage types require DDB2 for efficient repair [26,27]. Thereby, the primary function of the GG-NER damage sensing complex is detection of DNA structures, suspicious for the presence of modified nucleotides, rather than binding directly to the modification. Proper damage recognition takes place during subsequent steps by the transcription factor IIH (TFIIH) complex [9,28], which is activated for scanning of DNA for damage by another essential NER component XPA [29,30]. By all evidence, it is the XPD helicase within the TFIIH core—assisted by XPA and stimulated by the associated endonuclease XPG [30,31]—that plays the key role as the true damage recognition factor in a mechanism conserved across the kingdoms of life [9,32,33,34,35].

Since scanning of the damaged DNA strand by XPD takes place in the 5′-to-3′ direction, both the position and orientation of the TFIIH loading relative to the damage site are critical for damage recognition [36]. This is assured by XPC interaction with the p62 subunit of TFIIH [37]. Once TFIIH is engaged with DNA, activation of its XPB DNA translocase leads to helix opening and XPD loading on the damaged strand 5′ to the modification [38,39], which is guided by the co-assembling XPA, with its β-hairpin domain positioned at the junction between single-stranded and double-stranded DNA [9]. In addition to its essential scaffold function, XPA is required for the activation of the XPD helicase within TFIIH by displacing the regulatory trimeric MAT1-CDK7-Cyclin H kinase subcomplex [29,30]. The active TFIIH complex (further referred to as “Core7”) comprises, in addition to XPB and XPD, five other subunits—p62, p52, p44, p24, and p8/TTDA—all essential for the NER reaction [40]. According to the most current models, the ultimate damage recognition event is mediated by a stable interaction of the Arch domain of XPD stalled at the lesion, with some variations depending on the type of DNA lesion [41,42,43]. However, other interaction modes, specific to the damage structure, have been described as well and can be of a functional significance [44]. As modeled with DNA containing a bulky Cy5 modification opposite to a TpT dinucleotide, once XPD stalls at the damage site, the enduring XPB activity within the TFIIH Core7 complex leads to the DNA duplex opening by pushing it against W175 within the XPA β-hairpin, thus generating a pre-incision bubble around the damage site [9]. At the bubble size of approximately 30 nucleotides, coordinated incision takes place by the XPF (recruited to XPA via the interaction with its dimerization partner ERCC1) and XPG nucleases [45,46]. The GG-NER mechanism, including steps beyond damage recognition, is described in detail in the dedicated reviews [6,8,47,48,49,50].

**Figure 1:**
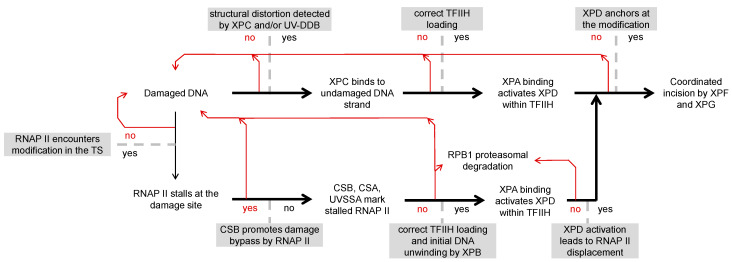
Scheme of the key events between the GG-NER (above) and TC-NER (below) damage recognition pathways and of the relevant decision points (grey boxes), as explained in the text. In both damage recognition modes, initial detection of the modified DNA structure is followed by stabilization of the damage binding protein complex, necessary for the TFIIH recruitment to a right position on the damaged DNA and in the right orientation; however, the mechanisms of these transitions are entirely different between GG-NER and TC-NER. The subsequent requirement for the local DNA helix melting by XPB is common for the two pathways, but XPA recruitment and binding at the DNA strand junction may not proceed via exactly the same mechanism, even though the role of TFIIH in this process is common between GG-NER and TC-NER. Although XPD loading on the damaged strand and activation by XPA are common, these steps are likely influenced by the presence of the stalled RNAP II complex, possibly depending on the mode of stalling at different types of DNA modifications. It is only after RNAP II displacement that the two pathways truly converge, with XPD as the ultimate damage recognition factor. Multiple experimental evidence implies that the pathways choice is to a major degree defined by the rate of binding of the GG-NER components XPC and UV-DDB as a default damage detection mechanism. Yet, it is intriguing to speculate that either the GG-NER or TC-NER process can abort at any of the listed critical steps and restart from a new damage detection round.

Before describing the damage detection mechanism by TC-NER, it is useful to note that GG-NER and TC-NER reactions converge starting from the XPA binding step and the resulting XPD engagement with the damaged DNA strand (Figure 1), implying an identical range of substrates recognized by the two pathways at the subsequent stages [7]. However, efficiencies of detection of a given DNA modification are not necessarily the same between the initial XPC binding and the final recognition by the XPD scanning—two steps within the GG-NER pathway. Indeed, many structures efficiently bound by UV-DDB and XPC/RAD23, as for instance the apurinic–apyrimidinic lesion within unpaired DNA stretches [25,51], are poorly recognized by the XPD helicase [28]. Conversely, even though large molecular size (“bulky”) DNA adducts are expected to efficiently halt the XPD helicase, a broad variety of them are poor substrates for the overall GG-NER reaction—either because of inefficient binding by XPC/RAD23 or due to an unproductive XPC alignment, which precludes correct loading of TFIIH [10]. It is remarkable that some of the identified GG-NER–resistant DNA modifications can apparently be removed by TC-NER, as reviewed in subsequent sections.

## 3. Molecular Mechanisms of Damage Sensing in TC-NER

According to current understanding, mammalian TC-NER differs from GG-NER only, or at least primarily, in the initial damage detection step. The discovery of the selective repair of the template DNA strand of transcribed protein-coding genes has immediately suggested a primary role of RNA polymerase (RNAP) II as the damage sensor within the TC-NER pathway, which was richly supported by subsequent research, as reviewed in detail elsewhere [7,12,52,53,54,55]. TC-NER substrates cause stalling of the elongating transcription complexes, which is characterized by stable association of RNAP II and the nascent transcript with damaged template DNA. In the case of the most extensively investigated TC-NER substrate, CPD, RNAP becomes arrested precisely at the damage site, with modification accommodated in the active site [56]. Other types of DNA damage cause stalling already a few nucleotides upstream of the modification site, as reported for the cisplatin-induced 1,2-GpG intrastrand crosslink [57].

Recognition and stabilization of damage-stalled RNAP II are the functions of the Cockayne syndrome B (CSB) protein, coded by the *ERCC6* gene. CSB is a SWI/SNF-related ATPase functioning in transcription as a chromatin remodeling and RNAP II processivity factor [58,59,60]. CSB dynamically associates with RNAP II during normal transcription of protein-coding genes, and this interaction is stabilized upon RNAP stalling [61,62]. While CSB activity would normally stimulate forward translocation of the transcription elongation complex, its function in the presence of a transcription-blocking entity on the template DNA is to stabilize the arrested conformation by preventing RNAP retrograde movement, commonly referred to as “backtracking” [58,63]. Structural studies with purified proteins have elucidated that this mechanism is conserved between human CSB and its yeast ortholog Rad26 [64,65]. Stabilization of the damage-stalled RNAP complex with CSB is the first step essential for the initiation of TC-NER. Accordingly, *ERCC6* was the first human TC-NER gene identified, based on its capacity to rescue the UV-sensitive phenotype of Cockayne syndrome complementation group B [16].

The exposed CSB motifs within the CSB-RNAP II complex recruit the CRL4^CSA^ ubiquitin E3 ligase through interaction with its CSA component, encoded by the human *ERCC8* gene [64,66]. Like CSB, CSA is essential for preferential repair of the template DNA strand, and its deficiency has been implicated in Cockayne syndrome [17,67]. The engagement of CSA and the associated ubiquitin ligase complex is of at least dual importance—it promotes ubiquitylation of lysine 1268 of the large RNAP II subunit RPB1 [68,69] as well as recruitment of UV-stimulated scaffold protein A (UVSSA) [64,66]. UVSSA binding is mediated by the N-terminal CSA-interacting region (CIR) and is enhanced and stabilized in the presence of CSB [66,70] and of the RPB1 ubiquitylation [68,71]. UVSSA protects CSB from degradation upon UV damage [70,72,73] and improves recovery of RPB1 protein levels in damaged cells, which was initially attributed to its association with ubiquitin-specific protease USP7 [70,72,73]. This mechanism could be of importance at least at high damage loads, when both CSB and RPB1 are targeted to proteasomal degradation by CSA-dependent ubiquitylation. Even more significant are reported interactions of UVSSA with TFIIH components [70,71]. In particular, the interaction of UVSSA amino acid residues 400–418 with the p62 pleckstrin homology (PH) domain is of crucial importance for TFIIH recruitment, as subsequently elucidated by several independent studies [66,68,74].

In summary, the coordinated assembly of CSB, CSA, and UVSSA is essential for TFIIH recruitment to the TC-NER damage recognition complex (Figure 1). Other proteins implicated in either TC-NER or the response to stalled RNAP II are STK19 and ELOF1 [75]. ELOF1 is necessary for UVSSA recruitment and enhances preferential repair of transcribed genes’ strands genome-wide by facilitating CRL4^CSA^-dependent RPB1 K1268 ubiquitylation [76,77]. STK19 stimulates the RPB1 ubiquitylation as well, additionally promoting correct binding of UVSSA [78,79,80,81]. The most important function of STK19 within the TC-NER complex is, however, the handover of the damaged DNA strand to TFIIH, wherein TFIIH is initially recruited via its p62 interaction with UVSSA, then brought 5′ from the damage site by the XPD interaction with STK19, and thus anchored for the XPB-dependent DNA helix opening and correctly positioned for XPD loading on the damaged DNA strand [78]. Functions of other proteins interacting with either damage-stalled RNAP II or the components of the downstream TC-NER complex, including the XPA-binding protein XAB2 [71,82], have yet to be elucidated.

Once both XPB and XPD are correctly engaged with damaged DNA, TFIIH transition to its damage-scanning mode would require displacement of RNAP II, which obstructs access to the modification site on the template strand. RPB1 poly-ubiquitylation followed by degradation is a common response to prolonged stalling or arrest under the conditions of induced transcription stress. However, RNAP II backtracking on the template with a possibility to resumption of transcription or eviction by dissociation from the DNA template would be more energetically favorable options for the cell [7,12]. Indeed, the proteasomal degradation pathway is essential for the clearance of damage-stalled RNAP II in the absence of UVSSA but is not required for its displacement in fully TC-NER–proficient cells [83]. A recent molecular modeling study has suggested an integrative mechanism for RNAP II dislodgement, initiated by recruitment of XPA to the TC-NER complex. As in GG-NER, XPA binding to the XPD Arch domain activates TFIIH by displacing the MAT1 inhibitory subunit. According to the proposed model, MAT1 removal concomitantly destabilizes STK19, which facilitates subsequent RNAP II dissociation by XPD pulling the damaged strand and CSB displacement by XPG binding to the upstream DNA [84] (Figure 1).

## 4. Available Methodology for Detection of TC-NER

### 4.1. Peculiarities of TC-NER Detection Compared to Pathways Active Genome-Wide

**Figure 2:**
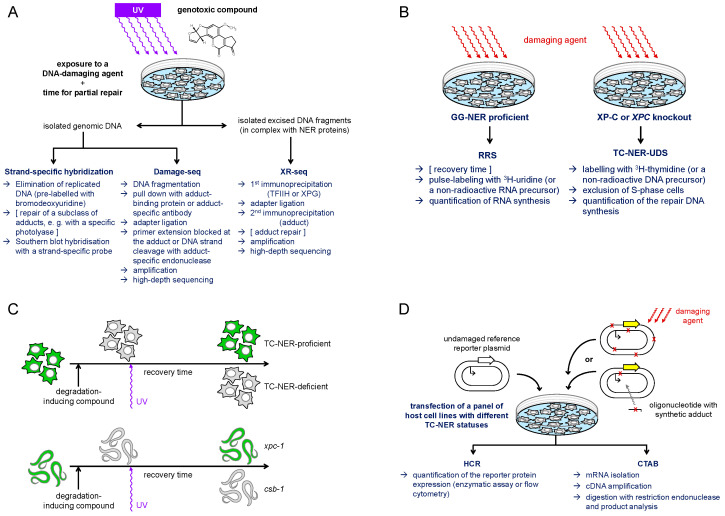
Summary of methodological approaches used for detection of TC-NER in mammalian cells. See main text for details. (**A**) Selected procedures for detection of the strand-selective-repair in genomic DNA. Southern blot hybridization with strand-specific probes as well as damage-seq detect the residual damage, whereas XR-seq and the related techniques detect the excised strand fragments. (**B**) The most commonly used indicator tests, based on the recovery of RNA synthesis (RRS) after the damage or on the TC-NER–specific unscheduled DNA synthesis (TC-NER-UDS). The latter assay is specific to TC-NER only if performed in a GG-NER–deficient cell model. (**C**) A protein synthesis recovery assay, based on the re-expression of an inducibly degradable reporter protein. The assay was proposed as a substitution for RRS, allowing real-time analyses of the gene expression recovery in cultured cells and entire organisms [90]. (**D**) Detection of repair of transcription-blocking damage in transfected plasmid DNA—the host-cell reactivation “HCR” and competitive transcription and adduct bypass (CTAB) assays. Both methods require a reference undamaged transcription template (co-transfected plasmid or a different gene on the same plasmid). The damaged gene can contain multiple modifications at random sites, generated by exposure to a genotoxic agent, or a defined DNA modification at a specific position in the transcribed strand. The latter is preferable for HCR and indispensable for CTAB.

Since the excision of DNA lesions by the NER pathway releases a 24–32-nt oligonucleotide, the resulting gap needs to be filled by synthesis of a relatively long patch of new DNA. Consequently, the repair patch synthesis, measured by incorporation of radioactive or otherwise labeled deoxyribonucleoside triphosphates into nuclear DNA outside of the S-phase, provides a sensitive and reliable indicator of NER efficiency in a cell line or a specifically damaged area in the nucleus [85,86,87,88]. However, in fully repair-proficient cells, only a small fraction of such “unscheduled” DNA synthesis (UDS) is attributable to TC-NER, because the great part of repair activity towards modifications induced genome-wide by most DNA damaging agents is provided by GG-NER. Even for UV radiation, the damaging agent used most commonly for the TC-NER research, GG-NER contributes to some 90% of UDS and thus can provide reliable measure of TC-NER only at a GG-NER deficient genetic background, e.g., in the absence of a functional *XPC* gene [89]. For the same reason, most of the standard methods for direct quantification of DNA damage and its removal from the genome (including chromatography coupled with mass-spectrometry, comet assay, or the antibody-based methods) cannot be applied as long as they do not discriminate between DNA strands. Therefore, a rather particular array of methods has emerged towards specific detection of TC-NER (Figure 2). All assays described below have technical pitfalls and limitations in the extrapolation of results to biologically relevant damage conditions in the genome. Nonetheless, rational integration of results deduced based on different endpoints allows their interpretation with a reasonable degree of confidence, especially when high-quality experimental data independently generated through different assays is available.

### 4.2. TC-NER Detection in Damaged Cells

#### 4.2.1. Strand-Specific Detection of DNA Damage Removal

TC-NER, defined as a preferential repair of the template strand of an actively transcribed gene, was originally discovered in the dihydrofolate reductase (*DHFR*) gene, amplified in methotrexate-resistant cell lines (human 6A3 and Chinese hamster ovary B11), following the damage induction by UV irradiation [18]. To measure strand-specific repair, DNA was incised with T4 endonuclease V, which cleaves DNA strand at the 5′ thymine or cytosine of the UV-induced cyclobutane pyrimidine dimers (CPDs), and subjected to Southern blot hybridization with strand-specific probes (Figure 2A). A clear advantage of such a hybridization-based assay is its applicability to the assessment of damage, induced in the genome of living cells and being processed under close-to-physiological conditions. A substantial limitation, on the other hand, is the requirement of a specific excision repair enzyme, which is not available for many types of DNA modifications of potential interest. A broad substrate specificity *Escherichia coli* UvrABC complex can be used if an actional repair approach for unwanted background modifications is available, such as pre-treatment with photolyase to repair CPDs by a direct damage reversal mechanism [91]. However, this adds another delicate step to the already laborious procedure, including the need to eliminate replicated DNA (bromodeoxyuridine labeling followed by density separation) as well as non-trivial preparation of strand-specific hybridization probes. It is not surprising, therefore, that the first identified TC-NER pathway components, CSB and CSA, were cloned using quicker and simpler, even if less precise, indicator assays that will be subsequently discussed [16,17].

#### 4.2.2. Transcription Recovery After Damage

UV irradiation of cells causes a rapid cessation of RNA synthesis. While RNA synthesis is restored in normal cells and in the XP cells deficient in GG-NER within hours after damage, skin fibroblasts from CS patients display a characteristic defect in the recovery of global transcription [92]. The impaired recovery of RNA synthesis (RRS) after UV damage was subsequently proved valuable for diagnosis of CS [93,94] and, accordingly, gained broad application as an indirect test for TC-NER deficiency (Figure 2B). More recent developments of non-radioactive RRS procedures towards automated imaging, combined with the use of alkyne-conjugated 5-ethynyuridine (EU) to replace ^3^H-uridine, make this assay easily adaptable at standard lab settings and suitable for high throughput screens [95]. In addition, protein synthesis recovery (measured either by immunoblotting or by fluorescence imaging techniques) has been proposed as a proxy for RRS (Figure 2C), which is particularly advantageous for application in tissues of living organisms, such as *Caenorhabditis elegans* [90].

RRS-based assays are indicative of TC-NER under the assumption that transcription restart is only possible after the removal of transcriptionally blocking DNA lesions. Indeed, RRS results demonstrate a good correlation with TC-NER repair efficiency and provide a reliable estimate for the severity of CS phenotype in the patients [16,94]. However, the assessed endpoint does not differentiate between molecular processes that are specifically involved in repair and those required for transcription restart. It is important to keep in mind that direct RNAP blockage at damage sites alone cannot account for global cessation of transcription in the nucleus, at least not within reasonably achievable genome damage loads. Indeed, the mechanisms of transcriptional inhibition by genotoxic agents are far more complex than simple blockage of elongating RNAP and the mechanisms of transcription restart after completion of repair largely have yet to be understood [7,12,96,97].

#### 4.2.3. Genome-Wide Mapping of Damage by Advanced Sequencing Techniques

Significant advancements in the understanding of TC-NER of several types of DNA modifications in the context of genome organization were provided by methods, named “damage-seq” and “eXcision Repair-seq” (XR-seq) (Figure 2A) [98,99]. The XR-seq procedure maps single-strand DNA fragments excised by NER to the genome. The oligomers ranging between 24 and 32 nucleotides length are released in complex with TFIIH [100], which shields them from degradation. Thus, the excised fragments can be isolated from the non-chromatin fraction and enriched by immunoprecipitation with antibodies specific to TFIIH components or to XPG [98,101]. To warrant specificity to a specific type of DNA adduct, another immunoprecipitation round is performed after the adapter ligation step. Also, it is necessary to remove adducts from the DNA prior to the amplification step, which in the case of UV-induced (6-4) photoproducts and CPD can be achieved with specific photolyases [98]. Alternatively, a suitable translesion synthesis-competent DNA polymerase can be used for library preparation in a modified procedure termed “tXR-seq” [102].

In damage-seq, genomic DNA is isolated from cells exposed to a DNA damaging agent of interest, fragmented by sonication, and immuno-enriched by an adduct-specific antibody, e.g., to a cisplatin GpG intrastrand crosslink. Specificity to damaged DNA is enhanced during the sequencing library construction by the choice of a DNA polymerase unable to bypass the specific type of damage [103]. Recently, damage-seq could be performed on DNA enriched for damage-stalled RNAP II by preceding chromatin immunoprecipitation with RPB1 antibody, providing information about the fate of damage-stalled RNAP II [83,104]. This procedure was referred to as “protein-associated DNA damage sequencing” (PADD-seq). A yet different principle termed “CPD-seq”, assisted by the cleavage with T4 endonuclease V in combination with apurinic/apyrimidinic endodeoxyribonuclease 1 (APE1), was used to map CPD lesions throughout yeast genome at single-nucleotide resolution [105]. However, since the analyses required extremely high UV doses (125 J/m^2^ UVC), the applicability of this method to mammalian cells has yet to be proven.

Damage-seq and XR-seq do not belong to routine genomic techniques and are difficult to establish in a lab, particularly due to tedious control steps required for the library preparation from damaged DNA. The requirement for exceptionally high sequencing depth, as well as necessity of analyzing multiple time points in cells recovering after genotoxic damage, makes these techniques extremely labor- and cost-intensive. In addition, a broader application of damage-seq and XR-seq is limited by the availability of antibodies, highly selective for specific DNA modification types. However, once these parameters are optimized, damage-seq and XR-seq are highly informative in providing the whole-genome information about damage generation and repair—at a single-nucleotide resolution, in a DNA strand-specific manner, and in the contexts of the mapped functional elements or chromatin features along chromosomes [99,106,107]. Applied either alone or in combination with damage-seq, XR-seq has provided clear indications for strand-specific repair of UV-induced CPD (in contrast to 6-4 photoproducts), with strand asymmetry enhanced in GG-NER–deficient XP-C cells and ablated in CS-B [98], which is in a perfect agreement with earlier reports about differential contributions of GG-NER and TC-NER to repair of these DNA lesions in selected single genes [18,91]. Other modifications displaying strand-specific repair based on XR-seq and/or damage-seq results are intrastrand crosslinks generated by the alkylating-like antineoplastic drug cisplatin at guanine dinucleotides (GpG) [99] as well as deoxyguanosine adducts of the fungal carcinogen aflatoxin B_1_ AFB_1_-N7-dGuo [107].

In addition to genome-wide mapping of damage, asymmetric distribution of characteristic signature mutations in NTS versus TS provides strong, although indirect, evidence for significant contribution of TC-NER to overall repair of DNA modifications that had originally caused the mutation. The challenges are that the observed mutational patterns can be ambiguously explained by either preferential repair in the TS or an increased damage in the NTS [108] and that many mutational signatures have yet to be assigned to the specific damaging agents [109]. Nonetheless, for DNA damage types with well characterized mutagenicity profiles, preferential accumulation of the characteristic mutations in the NTS should be interpreted as evidence for the preferential repair by TC-NER. For instance, strand asymmetry of the characteristic C>T and CC>TT transition mutations in melanoma skin cancers with respect to the direction of transcription can be assigned with a high confidence to UV-induced adducts at dipyrimidine sites containing cytosine [108,109,110].

### 4.3. Biochemical Reconstitution of TC-NER or of RNAP II Stalling

The complexity of the multistep TC-NER reaction described in Section 3, requiring coordinated recruitment and release of multiple protein components, makes its biochemical reconstitution particularly difficult. Until recently, only one report of TC-NER reconstituted with purified transcription and repair proteins was available, which demonstrated that TFIIH is recruited to RNAP II immobilized at a cisplatin-GTG intrastrand crosslink, followed by mobilization of the downstream NER components and leading to a CSB-dependent productive incision [111]. In addition to the technically demanding preparation of the transcription template with immobilized RNAP II, a major challenge is that NER components residing within the associated multiprotein complexes may co-purify during RNAP II isolation from mammalian cells [112]. This is a possible reason why TC-NER could occur in the initially described reaction despite the apparent absence of CSA and of other essential components that had been identified only later. The breakthrough in reconstituting TC-NER was achieved most recently by using transcription-competent *Xenopus laevis* egg nucleoplasmic extracts, immunodepleted for the XPC and supplemented with recombinant human TC-NER factors [78]. This frog egg extract-based system has not only provided extensive support to the mechanism proposed by the preceding computational structural studies (described in Section 3) but also helped to elucidate the function of STK19 as a newly identified TC-NER component.

It was early recognized in the field that the capacity of a specific type of DNA modification to arrest elongating RNAP complexes in cell-free transcription reactions is an excellent predictor for TC-NER. Classical TC-NER substrates—CPD and cisplatin intrastrand adducts—efficiently block various RNA polymerases in a DNA strand-specific manner [113,114,115]. Subsequently, transcription blocking capacities were extensively investigated using predominantly T7 RNA polymerase, various prokaryotic RNA polymerases, and RNAP II of either yeast or mammalian origin, as comprehensively reviewed in other publications [116,117,118]. Depending on the type of the lesion, the impacts on T7 RNA polymerase versus various multisubunit RNA polymerases (including RNAP II) are sometimes similar but can also be very different because of significant structural differences of the active site [115,119,120]. On the other hand, RNAP II enzymes from various eukaryotic sources—from yeast to human—have conserved structure of the active site and of the motifs involved in interactions with TC-NER components, allowing meaningful extrapolation of results between the species [117]. The obtained structures of RNAP II complexes stalled at the damage sites [56,57,121,122] were invaluable for current understanding of the molecular details of TC-NER.

The importance of RNAP II stalling for the initiation of TC-NER was corroborated by phenotypes of the engineered mutants of yeast Rpb1 (the largest RNAP II subunit), which have demonstrated that increased transcriptional bypass diminishes TC-NER, whereas transcription blockage enhances TCR of CPDs [123,124]. However, RNAP stalling alone should not be interpreted as proof of TC-NER activation. In addition to transcription blockage at the damage sites, RNAP stalling can also occur in the absence of DNA damage, without initiating TC-NER. It is necessary, therefore, for the cell to distinguish between spontaneous and damage-induced RNAP stalling, which appears to be the function of CSB [117].

### 4.4. Repair of Damage in Transfected Vector DNA

Plasmid-based expression vectors coding for reporter proteins can be conveniently used to determine repair competence of cells by transient transfection with damaged DNA, followed by measurement of the reporter protein expression [125] (Figure 2D). Before its use with naked UV-damaged plasmid DNA [126,127], a similar approach was initially applied to reactivation of damaged viruses in the infected cells, hence the name “host cell reactivation” (HCR) [128,129]. An important advantage of the HCR approach is the possibility to achieve damage densities required for maximum sensitivity (one or a few damaged sites per transcription unit) without inflicting excessive damage to the genome or off-target damage to other cellular compartments, which cannot be avoided when whole cells are exposed to damaging agents. Thanks to this, HCR provides much higher sensitivity and specificity of repair detection in comparison to the hybridization-based methods or RRS. Applied to skin fibroblasts isolated from XP patients of various complementation groups, HCR of UV-damaged plasmid reporters readily detects the NER defect as impairment of the reporter gene expression recovery in comparison to reference cells from healthy donors [126,127]. Moreover, HCR is easily compatible with the use of repair enzymes to remove specific subtypes of DNA adducts prior to transfection, which led to discovery that TC-NER deficiency of CS cells selectively impairs the repair of CDP but not of (6-4) photoproducts, before this was reported by other methods [130]. Another discovery that resulted from an application of an HCR-based assay was the identification of the human *CSA*/*ERCC8* gene as a complementation factor in CS-A [17].

To further enhance the specificity of the HCR assays, methods were developed to specifically incorporate single structurally defined adducts at specific nucleotide positions in plasmid DNA [131,132,133,134,135,136,137,138]. Reporter vectors harboring specific types of synthetic DNA adducts are useful to characterize transcription blocking properties of specific types of DNA modifications in NER-deficient cells [139,140]. Also, they help to elucidate the relevant repair mechanisms, based on the dependence of the recovery of the gene expression upon specific DNA repair pathway components [141,142,143]. Quantitative gene expression analysis can be performed by either using a reporter protein readout, as in the standard HCR assay, or by assessment of the mRNA transcript yields and sequence, by an assay called “competitive transcription and adduct bypass” (CTAB) [141] (Figure 2D). The CTAB method is based on reverse transcription and PCR amplification of RNA produced by expression of a vector containing site-specifically incorporated DNA lesions and a competitor vector in the same reaction. This is followed by PAGE and LC-MS/MS analyses of short DNA fragments released from the restriction digestion of RT-PCR products.

By design, synthetic oligonucleotides containing a modification of choice can be incorporated into the transcribed strand of the assessed gene, which makes CTAB and HCR assays particularly well suited for the detection of TC-NER. This had allowed identification of several adduct types that strictly rely on TC-NER for their repair, including the N^2^-deoxyguanosine adduct (N^2^-AAF-dG) of the experimental carcinogen 2-acetylaminofluorene [142], 8,5′-cyclopurine adducts arising from reactions of radical species [140,141], as well as exocyclic 3,N^4^-ethenocytosine (εC) and 1,N^2^-ethenoguanine (εG), induced by a lipid peroxidation product 4-hydroxy-2-nonenal [144,145]. Requirement of TC-NER was also assessed for N3-carboxymethylthymidine (N3-CMdT) and O^4^-carboxymethylthymidine (O^4^-CMdT), using CTAB assay [146]. After siRNA-mediated downregulation of CSB in human 293T cells, a significant decrease in transcriptional bypass efficiency of O^4^-CMdT occurred, while only a minor effect was reported for N3-CMdT. Even though both modifications did not cause a complete transcription block in the NER-deficient XP-A cell line, with an estimated bypass level of about 20%, the effect of CSB on the transcription recovery suggests a likely contribution of TC-NER to the repair in cells, at least in the case of O^4^-CMdT. Considering a relatively narrow dynamic range of CTAB values (only a two-fold difference between NER-proficient and XP-A cells) and hence only minimal effects of CSB knockdown, more conclusive data would be required to judge how important TC-NER is for the repair of O^4^-CMdT and, perhaps, N3-CMdT.

## 5. Types of DNA Damage Repaired Preferentially or Exclusively by TC-NER

### 5.1. UV-Induced Dipyrimidine Adducts

A clear asymmetry of the repair kinetics between the template and non-template strands at transcribed genome loci is best documented for CPD, the most common type of intramolecular adduct induced by UV [147]. The initial finding of a strand-selective CPD repair in the *DHFR* gene, later confirmed at the whole genome level [18,98] and corroborated by preferential accumulation of the characteristic UV signature mutations in the NTS versus the TS [108,109,110], allowed establishment of CPD as an invaluable DNA damage model for the investigation of TC-NER. Even though a minor CPD bypass apparently takes place in yeast [123], CPD can be regarded as a nearly absolute block to mammalian RNAP II, and the structure of transcription complex stalled at a thymine–thymine CPD has been resolved in detail [56], providing important knowledge for further understanding of the TC-NER mechanism.

A comparison between CPD and the structurally different pyrimidine (6-4) pyrimidone UV photoproduct is particularly instructive for interpretation of the strand selective repair, or in other words, the preferential repair pathway choice between GG-NER and TC-NER, in the cellular context. Although both types of the UV photoproducts block RNAP II very efficiently [113,114,148], the (6-4) photoproduct does not display a strand selective repair in fully NER-proficient cell lines [91,98]. Importantly, however, a GG-NER defect in the XP-C line unmasks the strand selective repair of (6-4) photoproducts, resulting in a repair kinetics very similar to CPD in the TS and virtually no repair in the NTS [91]. These results indicate that XPC scans the whole genome and detects at least some damage types faster than RNAP II even in transcribed sequences, suggesting that GG-NER is the primary repair pathway also in transcribed DNA (Figure 1). They also imply that accelerated repair of the template DNA strand of actively transcribed genes is limited to a subset of DNA modifications that are poorly detected by XPC.

In NER reactions with mammalian cell extracts or purified NER components, a thymine–thymine CPD is excised far less efficiently than the (6-4) photoproduct [149], due to the relatively inefficient binding of the CPD-containing DNA duplex by XPC, as discussed above [21,23]. This agrees well with the presented explanation of the preferential repair of CPDs, in contrast to (6-4) photoproducts, in the TS of actively transcribed genes. Still, it is essential to note that, in the absence of TC-NER, a large fraction of CPD lesions in the TS is removed apparently by GG-NER rapidly enough to prevent the damage site obstruction by stalled RNAP II. Indeed, TC-NER–deficient cell lines derived from CS patients (both of CS-B and CS-A complementation groups) retain very significant repair capacities towards synthetic thymine–thymine CPD incorporated into the TS of a reporter gene [142]. Similarly, disruption of either *DDB2* or *CSA*/*ERCC8* gene in isogenic HeLa-derived cell lines confers relatively mild impairment of the repair capacity towards CPD in the TS, as measured by HCR, suggesting that both GG-NER and TC-NER quantitatively contribute to CPD repair in the TS [140]. In contrast, repair of some other modifications, including the 3-(deoxyguanosin-*N*^2^-yl)-2-acetylaminofluorene (N^2^-AAF-dG) adduct or intranucleotide cyclopurine adducts, reviewed in subsequent sections, is severely impaired by either *CSA*/*ERCC8* or *CSB*/*ERCC6* deficiency whilst unaffected by the loss of either *XPC* or *DDB2* [140,142].

In summary, both major types of UV-induced DNA adducts, CPD and (6-4) photoproducts, block elongating RNAP II with similar potencies and can be efficiently processed by TC-NER. However, TC-NER is irrelevant for the repair of (6-4) photoproducts from the genome, including TS of active genes, because GG-NER removes these modifications even faster than TC-NER in cells where both NER pathways are functional. Neither is TC-NER essential for the repair of CPD. However, because GG-NER of CPD is slower than that of (6-4) photoproducts, a fraction of the damage can be removed by TC-NER as a backup pathway (Figure 1). Accordingly, it is for purely kinetic reasons that the contribution of TC-NER is more significant in highly expressed genes and at short distances from the transcription start, where the detection probability by elongating RNAP II is higher [98,108,150]. In addition, it is intriguing to suggest that the TC-NER may gain importance at high damage loads, which could potentially oversaturate the GG-NER damage detection capacity.

### 5.2. Adducts Formed by Reactive Metabolites of Carcinogenic Chemicals and Toxins

The example of the UV photoproducts illustrates that the importance of TC-NER for the repair of every particular damage type is co-determined by the GG-NER efficiency. NER reactions reconstituted with various structurally different adducts of chemical and dietary carcinogens revealed vast heterogeneity in the GG-NER excision efficiencies [151,152,153,154,155,156]. A comprehensive analysis of structural determinants rendering DNA lesions resistant to GG-NER has led to the identification of several classes of such modified nucleobases [10]. Although TC-NER efficiency has been determined only for a few of them, the modifications resisting GG-NER are obvious candidates for preferential or exclusive repair by TC-NER, provided that they block RNAP II, allow TFIIH loading and activation on the proper DNA strand, and can be recognized by XPD in the damage-scanning mode (Figure 1).

**Figure 3:**
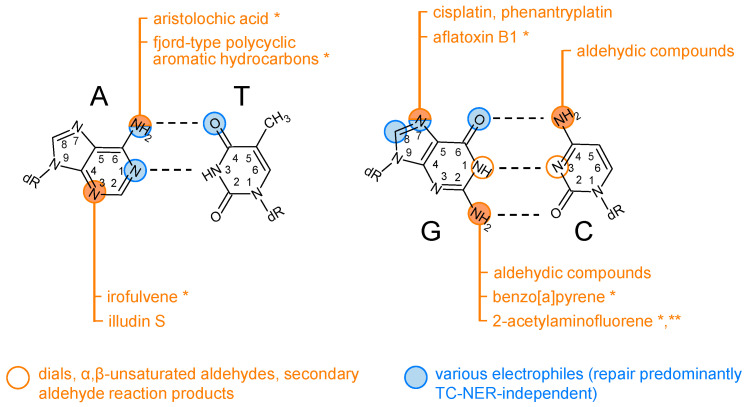
A map of positions at A:T and G:C base pairs, involved in the formation of adducts with alkylating-like drugs, aldehydes, and electrophilic metabolites of chemical or natural carcinogenic compounds, reported as likely TC-NER substrates by several independent assays. In all documented cases, RNAP II stalling requires a substituent larger than a methyl group. Blue circles indicate positions where putative TC-NER substrates can be measurably removed by concurrent DNA repair pathways (GG-NER, BER, or a direct damage reversal mechanism). Amber circles indicate positions where TC-NER is the vastly predominant or the only possible repair pathway, with relevant damaging agents listed. Marked with asterisks: (*) damaging agents requiring metabolic activation by cellular enzymes; (**) agents forming multiple adduct types, of which only an adduct found in a minor proportion requires TC-NER. Adducts formed by some bifunctional reactants (such as dials, α,β-unsaturated aldehydes, and secondary aldehyde adducts) can adopt ring-closed “exocyclic” forms, undergoing reversible ring opening at positions shown by open amber circles.

One structural class of GG-NER–resistant modifications is represented by N^6^-dA adducts of the fjord-type polycyclic aromatic hydrocarbons (PAH) benzo[c]phenanthrene [152,157] and dibenzo[a,l]-pyrene [10], which enhance base-stacking interactions by intercalating from the major groove, without inducing a base displacement. Another mechanism of NER avoidance by PAH adducts is illustrated by strong isomer-specific differences of the excision of benzo[a]pyrene-N^2^-dG [10,151,154]. Two of the four possible adduct types (the *trans*-isomers) are accommodated in the minor groove, without causing a significant structural distortion or thermodynamic destabilization of the double helix and being poor GG-NER substrates. In contrast, the *cis*-adducts adopt base-displaced intercalative conformations, causing extrusion of both the modified and its partner base, which is efficiently recognized by the XPC protein, resulting in a very efficient NER [10,151,158]. Such a productive XPC binding is apparently hindered by PAH adducts in the minor groove conformation, independently of the adduct size, or by intercalation in the absence of base displacement, as in the case of fjord PAH [10]. It is, therefore, expected that at least a subset of PAH adducts should require TC-NER for the efficient repair in cells. In support of the hypothesis about TC-NER as a prevailing repair pathway requirement for the N^2^-dG adducts (Figure 3), cell cultures exposed to benzo[a]pyrene accumulate predominantly C>A mutations, with a significant strand bias towards the non-transcribed strand G [159,160,161,162,163]. However, the benzo[a]pyrene diol epoxide (BPDE) adduct did not show a clear strand preference in the tXR-seq assay, perhaps due to insufficient sequence depth as well as high BPDE cytotoxicity [102].

Another important class of DNA adducts requiring NER arises from exposure to aromatic amines, including heterocyclic aromatic amines formed in protein-rich food by cooking. Their electrophilic metabolites form adducts preferentially at C8-dG and to a lesser degree at N^2^-dG, as illustrated by the toxification of the experimental carcinogen 2-acetylaminofluorene [164]. The structures of the modified DNA duplexes containing *N*-(deoxyguanosin-8-yl)-2-acetylaminofluorene (C8-AAF-dG) and 3-(deoxyguanosin-*N*^2^-yl)-2-acetylaminofluorene (N^2^-AAF-dG) have revealed striking differences between the two modification types. The presence of C8-AAF-dG in DNA results in a large degree of helical distortion and significant thermodynamic destabilization of the duplex by assuming a base-displaced intercalative conformation [165], making it an excellent substrate for the reconstituted NER reactions [45,151,155]. Also in the cellular context, C8-AAF-dG removal from TS of the reporter gene occurred by GG-NER in CS-A and CS-B cells as efficiently as in a TC-NER–proficient cell line [142].

In contrast, N^2^-AAF-dG is accommodated in the minor groove of the helix [166], similar to the subclass of BPDE adducts discussed above [10]. As measured by HCR in several independently generated isogenic human cell lines, N^2^-AAF-dG is entirely resistant to GG-NER. Moreover, reporter constructs carrying a single synthetic N^2^-AAF-dG adduct show powerful RNAP II transcription blockage in the absence of TC-NER as well as absolute requirements for *CSA* and *CSB* for the gene expression reactivation [142,145]. This conclusion is corroborated by the adduct removal kinetics in cultured rat hepatocytes, where N^2^-AAF-dG adduct (allegedly removed only from the transcribed portion of the genome) was found to be particularly persistent in contrast to C8-AAF-dG [167]. A similar persistence of the N^2^-dG adduct in contrast to the C8-dG counterpart was independently reported in rat organs after exposure to the heterocyclic 2-amino-3-methylimidazo[4,5-f]quinoline [168]. Combined, the presented evidence not only highlights N^2^-AAF-dG as an obligatory TC-NER substrate but also suggests that the requirement of TC-NER might be a common feature on N^2^-dG adducts accommodated within the minor groove (Figure 3).

Metabolism of some plant and fungal toxins can lead to reactive compounds that cause DNA adducts, structurally similar to GG-NER–resistant lesions generated by the carcinogenic chemicals described above. One example is the natural plant compound aristolochic acid, metabolized to aristolactam and the derived nitrenium ion, which reacts preferentially with N^6^-dA in DNA. The resulting adducts resemble the structure of the fjord-type PAH adducts [169,170]. At least a subclass of these adducts is relatively resistant to GG-NER [171]. Moreover, the A>T mutational signature of aristolochic acid in cancers has a pronounced overrepresentation of A in the non-transcribed strand [172,173,174], suggesting at least a preferential repair by TC-NER (Figure 3). A similar enrichment of mutations originating from the non-transcribed strand is reported for the carcinogenic mycotoxin aflatoxin B_1_ (AFB_1_), whose epoxydic metabolite reacts preferentially at N7-dG, leading to several DNA modifications, depending on the chemical decay pathway of the primary adduct [175]. At least a subclass of damage generated by AFB_1_ is excised preferentially from the transcribed strand of active genes, as determined by XR-seq [107]. The strand selectivity of the repair is reflected by the transcription strand bias of the characteristic AFB_1_-induced G>T mutation in liver tumors [162,176], strongly suggesting that some type of damage induced by AFB_1_ is preferentially removed by TC-NER (Figure 3).

### 5.3. Adducts Formed by Alkylating(-like) Agents

Like metabolized AFB_1_, several anticancer drugs, including cisplatin, react preferentially at N7-dG, forming, differently from AFB_1_-N7-dG, chemically stable adducts. Thus, a bifunctional alkylating-like agent cisplatin generates the GpG, ApG, and GpNpG diadducts on the same DNA strand and, at lower frequency, monoadducts as well as highly cytotoxic interstrand G-G crosslinks [177]. Of the two structurally different types of the intrastrand crosslinks involving guanosines, cisplatin-GpG is excised by GG-NER far less efficiently than the GpNpG crosslink [178], while both adducts block RNAP II with high efficiencies [115]. Accordingly, the 1,2-GG adduct should be regarded with a high likelihood as a TC-NER–specific substrate (Figure 3). This is strongly supported by mutation analyses of secondary malignancies in patients treated with cisplatin for their primary cancer: the analyzed tumors contained characteristic single nucleotide C>T substitutions as well as dinucleotide substitutions with a pronounced enrichment of G in the non-transcribed strand [109,179]. Moreover, both XR-seq and DNA damage-seq analyses in a human lymphocyte cell line revealed preferential NER of the GpG cisplatin adduct in the transcribed DNA strand, providing no such evidence for GpNpG sequences. Finally, in a cell-free NER system reconstituted with nucleoplasmic extracts of *X. laevis* eggs, the 1,3-GpTpG intrastrand crosslink on the transcription template strand was efficiently repaired in an XPC-dependent manner, both in the absence and in the presence of transcription [78]. Combined, these results convincingly indicate that that cisplatin GpNpG are very efficiently detected and repaired faster by GG-NER than by TC-NER. In contrast, repair of the GpG adduct heavily relies on TC-NER. This importance of TC-NER is reflected in the increased sensitivity of cells with disrupted TC-NER components to cisplatin, identifying another cytotoxicity mechanism of this compound in addition to the pathway assigned to the interstrand crosslinks [75].

A fungal sesquiterpene compound illudin S and its semisynthetic derivatives acylfulvenes, the latter upon metabolic activation by endogenous prostaglandin reductase 1, react with DNA by addition predominantly at position 3 of adenine [180,181]. Despite an expected rapid loss of the resulting N3-alkyl-dAdo adducts from DNA by spontaneous depurination [182,183], compromised TC-NER significantly sensitizes mammalian cells both to illudin S and acylfulvenes. Thus, cells deficient in either CSA or CSB show extreme sensitivity to illudin S as well as impaired UDS, comparable to the phenotype of XP-A cells with a total NER defect [184]. Similarly, CS-B and XP-A cells display equivalent increase in cytotoxicity as well as impaired RRS in response to acylfulvene compounds [185,186]. TC-NER requirement for the repair of the illudin S-induced DNA damage was independently confirmed by an unbiased chemogenomic CRISPR-Cas9 screen, which pinpointed all core NER genes as well as all thus far known TC-NER components, including then newly identified ELOF1 and STK19, as protection factors against illudin S [75]. Another independent confirmation of TC-NER requirement is provided by a pronounced strand bias of the characteristic illudin S mutational signature, dominated by T>A transversions, and a clear inverse correlation of the observed mutation frequencies with the gene expression levels [187]. Unfortunately, due to the intrinsic instability of the N3-alkyl-dAdo adducts, capacities of the illudin S or acylfulvene adducts to block RNAP II could not be tested directly. However, their stable analog 3-deaza-3-methoxynaphtylethyl-adenosin caused a robust RNAP II blockage, and the crystal structures of stalled yeast RNAP II were obtained, suggesting a mechanism involving the amino acid residues interacting with the minor groove of the DNA:RNA hybrid in the posttranslocation position [188]. Intriguingly, several adducts affecting the minor groove interface of other nucleobases were also implicated in transcriptional blockage, as discussed in the following section.

A very special case of a natural compound reacting at N^2^-dG via an electrophilic intermediate is the tunicate alkaloid trabectedin, also known as “ecteinascidin 743” (Et743), whose toxicity is exacerbated by active TC-NER [189,190,191]. The covalent Et743 adduct causes DNA to bend toward the major groove, which leads to a strong double helix stabilization, thermodynamically comparable with the effect of an interstrand crosslink [192,193]. This feature would explain resistance of the Et743 adducts to GG-NER, by analogy with other minor groove adducts discussed above. However, the truly remarkable property of Et743 to hijack NER nucleases via a TC-NER damage recognition mechanism has yet to be fully explained. Based on the capacity of the Et743 adducts to block RNAP II in a strand-independent manner [194], it can be proposed that TFIIH loading and subsequent incision could be directed to a wrong DNA strand. It is also conceivable that premature XPD stalling by stabilized base pairs in the proximity to the adduct may lead to misalignment of the XPG and XPF/ERCC1 nuclease complexes and a futile incision.

### 5.4. Adducts Induced by Aldehydes

The N^2^ position of guanine does not belong to major targets for reactions of alkylating agents with DNA. However, N^2^-alkyl-deoxyguanosine (N^2^-alkyl-dG) adducts can arise via a reduction of the primary adducts generated at this position by reactive aldehydes. For instance, N^2^-ethylidene-2′-deoxyguanosine, which is induced in the genome by exposure to acetaldehyde, undergoes reduction to N^2^-ethyl-2′-deoxyguanosine (N^2^-ethyl-dG) [195]. Whilst many types of DNA adducts induced by aldehydes are unstable and thus hard to analyze, their structurally similar chemically inert alkyl analogs are useful for understanding of the relevant repair pathways. N^2^-ethyl-dG is a strong block to RNA polymerases, including mammalian RNAP II [119].

N^2^-ethyl-dG is strongly transcription-blocking in the cellular context as well, as analyzed by HCR in NER-deficient XP-A cells [145]. In this system, the inhibitory effect of N^2^- ethyl-dG on the reporter gene expression was specific to the template DNA strand, and the recovery of transcription was strictly dependent on *CSA* and *CSB*, but not *XPC* or *DDB2*/*XPE*, which strongly suggests that repair of this modification requires TC-NER (Figure 3). Remarkably, a smaller adduct, N^2^-methyl-dG, did not have a detectable impact of the transcriptional output, whereas the magnitude of transcriptional blockage by N^2^-ethyl-dG in the template DNA strand was equivalent to the effect of a much bigger N^2^-AAF-dG, suggesting that an efficient transcription blockage requires a minimal adduct size; however, the capacity to cause RNAP II stalling does not increase proportionally to the adduct bulk once the critical substituent size (in this case, ethyl group) has been reached. Since N^2^-methyl-dGuo does not block transcription to any significant degree (Figure 3), its recognition by TC-NER is unlikely. Another interesting observation was that, despite quantitatively similar transcription-blocking capacities, the bulkier N^2^-AAF-dG showed significantly higher reactivation rates by TC-NER than N^2^-ethyl-dG, which could reflect a more efficient handover of the damaged strand to TFIIH or a better recognition by XPD within the TFIIH complex.

To understand the mechanism of transcription blockage by N^2^-ethyl-dG, it is useful to compare it with other ethylated modifications, such as O^2^-, N3-, and O^4^-ethylthymidine (O^2^-, N3-, and O^4^-ethyl-dT), induced by metabolic activation of *N*-nitroso compounds in the cells. These modifications inflicted variable degrees of transcription stalling in transcription bypass reactions reconstituted with the *Saccharomyces cerevisiae* RNAP II, with O^2^-ethyl-dT being the most harmful [196]. Using structural modeling, the authors proposed a mechanism that O^4^-ethyl-dT is tolerated within the active site, whereas the ethyl group of O^2^-ethyl-dT may collide during translocation with P448 of the loop formed by the Rpb1 amino acid residues 440–460, which act as a “steric gate” for this minor groove-oriented adduct within the newly formed DNA:RNA hybrid. The findings parallel the behavior of the template N^2^-ethyl-dG paired with the incoming CTP, for which a remarkably similar mechanism was modeled previously [119]. Together, these findings suggest that an ethyl group faced towards the minor groove of the DNA:RNA duplex provides a sufficient structural determinant for damage sensing by RNAP II, being the common feature of dG:C and dT:A (DNA:RNA) pairs. Even though adducts smaller than the ethyl moiety were not tested at the O^2^ position of thymine and it yet remains to be proven whether O^2^-alkyl-dT undergoes TC-NER, the proposed mechanism provides a mechanistic rationale for the evidence of transcription blockage and transcription-coupled repair of N^2^-ethyl-dG and N^2^-AAF-dG (Figure 3), discussed in the previous paragraph.

Reactions of nucleobases with dials and α,β-unsaturated aldehydes yield a broad spectrum of so-called exocyclic DNA adducts [197]. At least two of the adducts of this class, 3,N^4^-ethenocytosine and 1,N^2^-ethenoguanine, have been characterized as preferential TC-NER substrates based on combined evidence of the RNAP II blockage as well as defective HCR and specific accumulation in the genome of TC-NER deficient cells [120,144,145] (Figure 3). A broad structural variety of exocyclic adducts can also be formed as a result of exposure to carcinogenic chemicals, such as vinyl chloride, but also by reactions of endogenously generated metabolites. Thus, various exocyclic adducts, including the mentioned 3,N^4^-ethenocytosine and 1,N^2^-ethenoguanine, can be generated among other pathways by peroxidation products of unsaturated fatty acids, induced either by metabolic stressors or endogenously arising reactive oxygen species [195,197]. Among different repair pathways being involved in the processing of this structurally heterogenous class of nucleobase adducts, the significance of TC-NER has yet to be assessed for the specific modifications.

### 5.5. Damage to Nucleobases Induced by Endogenously Arising Reactive Oxygen Species

**Figure 4:**
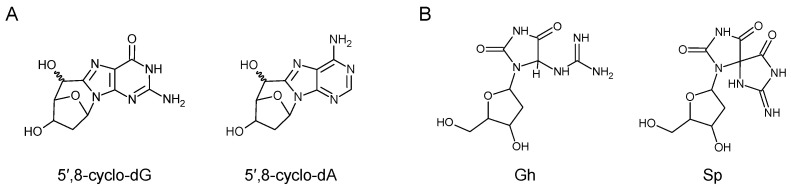
Chemical structures of oxidatively generated DNA modifications requiring TC-NER and/or causing RNAP II stalling as discussed in Section 5.5. (**A**) The cyclopurine lesions cyclo-dG and cyclo-dA arise in DNA under exposure to hydroxyl radicals. Both types of cyclopurine lesions efficiently block transcription in a strand-specific manner, and the TC-NER requirement for their repair is strongly supported by multiple independent lines of evidence. (**B**) Spiroiminodihydantoin (Sp) and 5-guanidinohydantoin (Gh) are advanced oxidation products that can be formed from 8-oxo-7,8-dihydroguanine. Sp and Gh are regarded as candidate TC-NER substrates, based on their capacities to stall RNAP II. However, there is also evidence of their processing by GG-NER and BER, at least under reconstituted cell-free conditions.

Endogenous sources of damage to DNA are of special concern in the TC-NER field, because pathological features of Cockayne syndrome, a severe disease caused by homozygous CSA or CSB mutations, are most pronounced in the brain—the organ typically most protected from the exposure to environmental and dietary toxicants. In addition to the damage induced by endogenous aldehydes, discussed in the previous section, reactive oxygen species arising from the aerobic metabolism may contribute to such damage, especially in the brain as the organ with the highest energy demand [198]. Among multiple types of nucleobase modifications generated by reactions with reactive oxygen species, a particular type of intranucleotide adducts, 8,5′-cyclo-2′-deoxyguanosine (cyclo-dG) and 8,5′-cyclo-2′-deoxyadenosine (cyclo-dA), each occurring as 5′R and 5′S diastereomers, are of a special interest (Figure 4A) [199,200].

Cyclopurine DNA adducts are intrinsically resistant to BER and can be repaired in cell-free NER reactions with far lower (40 or 150 fold) efficiencies than the cisplatin GNG intrastrand crosslink as a reference GG-NER substrate [201,202], essentially leaving TC-NER as the only conceivable repair pathway. Both cyclo-dG and cyclo-dA efficiently stall RNAP II under cell-free conditions [141] and potently inhibit reporter gene expression in NER-deficient XP-A cells in a manner, specific to the template DNA strand, strongly suggesting a RNAP-mediated transcription blocking mechanism [140,203]. Accordingly, structural studies in the yeast cell-free transcription system revealed close similarities between the mechanisms of the RNAP II stalling at the cyclo-dA and CPD lesions [117,121]. HCR of cyclo-dA and cyclo-dG in human cell lines is completely abolished by disruption of either *CSA* or *CSB*, indicating that TC-NER is the only available repair pathway for these lesions [145]. In agreement with the TC-NER requirement for repair, 8,5′-cyclopurine adduct levels are increased in *Csb^−/−^* mice [204]. Moreover, cyclopurine DNA adducts accumulate in organs of aging animals, and this accumulation is exacerbated by gene knockout of the core NER component *Ercc1* [205]. Also, cultured cells originating from Cockayne syndrome patients display accumulation of these lesions under hypoxic conditions—to a higher degree than cells complemented with functional *CSA* and *CSB* [206].

8,5′-Cyclopurine adducts can only arise by action of hydroxyl radicals (•OH) on DNA but not by other reactive oxygen species, suggesting that agents and biochemical pathways leading to intranuclear generation of •OH could be of a special concern for the pathogenesis of Cockayne syndrome and the neurological manifestations of Xeroderma pigmentosum in the patients with full NER deficiency [199,200]. Due to its exceptionally high reactivity, •OH generates a variety of DNA modifications [207], some of which, such as a well-characterized pyrimidine oxidation product thymine glycol (Tg), can be excised in cell-free NER reactions slightly less efficiently than CPD [208]. However, mammalian (rat) RNAP II efficiently bypasses Tg in the template DNA strand in the reconstituted transcription system [209]. In transiently transfected NER-deficient (XP-A) cells, reporter genes harboring a synthetic BER-resistant Tg analog were expressed at unaltered levels, regardless of the position of the modification in the template or the non-template DNA strand. The expression was only decreased when BER of Tg was enabled, in a strictly NTHL1-dependent manner and without any specificity to the template DNA strand [140]. These results thus exclude a significant TC-NER contribution to cellular repair of Tg and assign the negative effects on the gene expression to a post excision product generated by BER, akin to the mechanisms commonly described for other BER substrates [139,210,211,212].

The most common oxidatively generated nucleobase modification, 8-oxo-7,8-dihydroguanine 8-oxoG, should not be considered as a biologically relevant TC-NER substrate, despite some excision observed in the reconstituted GG-NER system [208]. Unlike 8,5′-cyclopurine adducts, 8-oxoG does not strongly block RNAP II [213,214] and does not interfere with gene transcription in mammalian cells, unless cleaved by OGG1 and APE1 in the process of BER [215,216]. However further 8-oxoG oxidation at the pyrimidine ring [217] can lead to products implicated in stalling of RNAP II, such as spiroiminodihydantoin (*R*-Sp and *S*-Sp) and, to a lesser extent, 5-guanidinohydantoin (Gh) [122] (Figure 4B). Thus, in experimental systems relying on DNA damage induced by reactive oxygen species, rather than synthetic modifications of a defined type, observation of TC-NER of Sp or Gh could be erroneously assigned to 8-oxoG. However, it remains to be established that RNAP II stalling at Sp and/or Gh leads to a productive TFIIH loading and whether TFIIH can detect these lesions by scanning the damaged DNA strand. In addition, both modifications are efficiently excised by GG-NER in an XPC-dependent manner and also by the DNA glycosylase NEIL1 under cell-free conditions [218,219]. Considering the availability of multiple repair pathways for Sp and Gh, the pathway choice in the cell remains an open question for these DNA modifications.

### 5.6. Can Apurinic/Apyrimidinic (AP) Lesions Be Processed by TC-NER?

Some of the DNA modifications discussed in the previous sections, including N3-dAdo adducts generated by illudin S and N7-dGuo adducts of aflatoxin B_1_, are prone to rapid (within hours) spontaneous hydrolytic depurination [183]. Whilst specific mutational signatures of these agents (A>T for illudin S and G>T for aflatoxin B_1_) demonstrate pronounced asymmetry between the TS and the NTS [162,176,187], it is a major question whether TC-NER is engaged at the stage of the primary adduct or does it contribute to the repair of AP lesions remaining after the departure of the damaged base. Even though AP lesions are efficiently recognized and cleaved by the specific apurinic/apyrimidinic endonuclease APE1 within the base excision pathway (BER) [220], the reactivity of the aldehydic group of deoxyribose may lead to the formation of modified forms of the AP lesion, resistant to APE1. Various stable synthetic analogs of the AP lesion are recognized in the reconstituted GG-NER systems [23,28,221], even though AP lesions do not pose a strong barrier to TFIIH helicases [28]. AP lesions induce only moderate RNAP II stalling in cell-free transcription reactions, which may be insufficient for TC-NER initiation [222,223]. Accordingly, only a mild decrease in the reporter gene expression was observed in NER-deficient XP-A cells transiently transfected with a construct containing an APE1-resistant AP site analog—tetrahydrofuran (THF) flanked by an uncleavable linkage on the 5′—in the template strand [139]. Because of the absence of a robust transcription block, the repair capacities towards the synthetic AP lesion in various NER-deficient cell lines were measured based on the rates of transcriptional mutagenesis rather than by the HCR principle. An efficient removal of the THF lesion by NER was detected; however, the NER repair capacity was largely retained in CSA- and CSB-deficient cells, suggesting that GG-NER was the major mechanism [139]. It remains to be established whether natural AP lesions or some of their reaction products arising in the cellular environment require TC-NER for their processing.

## 6. Current Advances and Future Directions

Comprehensive structural modeling of the dynamic organization of the TC-NER damage sensing complex up to the TFIIH loading step, elucidation of the role of the RPB1 K1268 ubiquitylation, identification of the new specific effector proteins STK19 and ELOF1, new insights into the CSB, CSA, and UVSSA functions, and successful biochemical reconstitution of all steps of the TC-NER reaction have remarkably advanced the field of TC-NER in recent years. These breakthrough findings and techniques conclusively confirm the central role of RNAP II as the primary damage sensing factor in TC-NER and underscore the validity of RNAP II stalling as useful endpoint for classification of a given type of DNA modification as a TC-NER substrate. However, to-date, TC-NER reactions were modeled only for a few types of DNA modification and damaging agents, raising the question of whether more than one mode of TC-NER may exist for different damage types. Even for the limited amount of DNA adducts analyzed so far, several structural modes for RNAP II stalling were described [117], and further research may reveal more. For instance, the extreme case of Et743, which causes RNAP II stalling in an unconventional manner and apparently leads to mistargeted incision, might reveal a distinct TC-NER modality in the future. Another intriguing direction to be explored within the TC-NER mechanism is whether the newly identified TFIIH interaction with RPB6, the common subunit of all three eukaryotic RNA polymerases, has a potential to target TC-NER to transcribed DNA sequences beyond the protein-coding genes [224]. Finally, the mechanism of TFIIH scanning and the ultimate damage recognition by XPD can also differ depending on the damage structure [35]. The structural determinants for the XPD-driven damage recognition are certainly important for the repair outcome and need to be understood in the future.

Another recent discovery has brought into evidence a cross-pathway role of the key TC-NER components *CSA* and *CSB*, both of which turned out to contribute to the repair of DNA–protein crosslinks (DPCs) and to play key roles in transcription recovery following formaldehyde-induced DPC formation [225,226,227]. CSA and CSB are recruited to DPC-stalled RNAP II, initiating the damage processing, which is at least partly independent from the RPB1 K1268 ubiquitylation and does not require UVSSA and XPA. It is, thereby, unlikely that the described mechanism includes DNA excision and resynthesis. Although clearly different from the conventional TC-NER pathway, the protection from cytotoxic transcription-blocking DPCs—impaired by the loss of either CSB or CSA—may explain the severe Cockayne syndrome features in patients of the respective gene complementation groups, contrasting with the mild phenotypes linked to known *UVSSA* mutations.

Beyond the open questions of TC-NER biochemistry, a continuous effort is necessary towards understanding TC-NER in the cellular context. A major obstacle for this research is limited specificity of the available damaging agents, which ideally should react only with DNA (like UV light) and induce strictly the types of modifications whose repair requires TC-NER (like illudin S). However, the damage profile of illudin S has been only partly characterized, and it should be kept in mind that UV irradiation of DNA induces a complex mixture of photoproducts, with GG-NER significantly contributing to the repair of CPDs and, especially, 6-4 photoproducts. The combination of techniques using synthetic DNA lesions (HCR ant CTAB assays) with computation-based deconvolution of mutational signatures in cells exposed to agents with known damage profiles has allowed the identification of TC-NER requirement for the repair of several types of DNA modifications reviewed here. This redefines the significance of TC-NER as the indispensable repair mechanism for selected types of DNA damage, rather than merely an accelerated version of NER. By arguing that the evidence of TC-NER is indicative of an inefficient repair genome-wide, we encourage a broader investigation of repair of defined of DNA modifications as well as assignment of the orphan mutational signatures (https://cancer.sanger.ac.uk/ (accessed on 9 July 2025)) to the specific modifications in the future—towards understanding which genotoxic insults lead to most persistent DNA damage, with potential implications for disease and aging.

## Data Availability

The original contributions presented in this study are included in the article. Further inquiries can be directed to the corresponding author.

## References

[1] Lindahl T. (1993). Instability and decay of the primary structure of DNA. Nature.

[2] Jiricny J. (2013). Postreplicative mismatch repair. Cold Spring Harb. Perspect. Biol..

[3] Krokan H.E., Bjoras M. (2013). Base excision repair. Cold Spring Harb. Perspect. Biol..

[4] Scharer O.D. (2013). Nucleotide excision repair in eukaryotes. Cold Spring Harb. Perspect. Biol..

[5] Vermeulen W., Fousteri M. (2013). Mammalian transcription-coupled excision repair. Cold Spring Harb. Perspect. Biol..

[6] D’Souza A., Kim M., Chazin W.J., Scharer O.D. (2024). Protein-protein interactions in the core nucleotide excision repair pathway. DNA Repair.

[7] Nieto Moreno N., Olthof A.M., Svejstrup J.Q. (2023). Transcription-Coupled Nucleotide Excision Repair and the Transcriptional Response to UV-Induced DNA Damage. Annu. Rev. Biochem..

[8] Kuper J., Kisker C. (2023). At the core of nucleotide excision repair. Curr. Opin. Struct. Biol..

[9] Kim J., Li C.L., Chen X., Cui Y., Golebiowski F.M., Wang H., Hanaoka F., Sugasawa K., Yang W. (2023). Lesion recognition by XPC, TFIIH and XPA in DNA excision repair. Nature.

[10] Geacintov N.E., Broyde S. (2017). Repair-Resistant DNA Lesions. Chem. Res. Toxicol..

[11] Llerena Schiffmacher D.A., Pai Y.J., Pines A., Vermeulen W. (2025). Transcription-coupled repair: Tangled up in convoluted repair. FEBS J..

[12] Lans H., Hoeijmakers J.H.J., Vermeulen W., Marteijn J.A. (2019). The DNA damage response to transcription stress. Nat. Rev. Mol. Cell Biol..

[13] Bukowska B., Karwowski B.T. (2018). Actual state of knowledge in the field of diseases related with defective nucleotide excision repair. Life Sci..

[14] Karikkineth A.C., Scheibye-Knudsen M., Fivenson E., Croteau D.L., Bohr V.A. (2017). Cockayne syndrome: Clinical features, model systems and pathways. Ageing Res. Rev..

[15] Marteijn J.A., Lans H., Vermeulen W., Hoeijmakers J.H. (2014). Understanding nucleotide excision repair and its roles in cancer and ageing. Nat. Rev. Mol. Cell Biol..

[16] Troelstra C., van Gool A., de Wit J., Vermeulen W., Bootsma D., Hoeijmakers J.H. (1992). ERCC6, a member of a subfamily of putative helicases, is involved in Cockayne’s syndrome and preferential repair of active genes. Cell.

[17] Henning K.A., Li L., Iyer N., McDaniel L.D., Reagan M.S., Legerski R., Schultz R.A., Stefanini M., Lehmann A.R., Mayne L.V. (1995). The Cockayne syndrome group A gene encodes a WD repeat protein that interacts with CSB protein and a subunit of RNA polymerase II TFIIH. Cell.

[18] Mellon I., Spivak G., Hanawalt P.C. (1987). Selective removal of transcription-blocking DNA damage from the transcribed strand of the mammalian DHFR gene. Cell.

[19] Sugasawa K., Ng J.M., Masutani C., Maekawa T., Uchida A., van der Spek P.J., Eker A.P., Rademakers S., Visser C., Aboussekhra A. (1997). Two human homologs of Rad23 are functionally interchangeable in complex formation and stimulation of XPC repair activity. Mol. Cell Biol..

[20] Sugasawa K., Ng J.M., Masutani C., Iwai S., van der Spek P.J., Eker A.P., Hanaoka F., Bootsma D., Hoeijmakers J.H. (1998). Xeroderma pigmentosum group C protein complex is the initiator of global genome nucleotide excision repair. Mol. Cell.

[21] Sugasawa K., Okamoto T., Shimizu Y., Masutani C., Iwai S., Hanaoka F. (2001). A multistep damage recognition mechanism for global genomic nucleotide excision repair. Genes. Dev..

[22] Zhang E.T., He Y., Grob P., Fong Y.W., Nogales E., Tjian R. (2015). Architecture of the human XPC DNA repair and stem cell coactivator complex. Proc. Natl. Acad. Sci. USA.

[23] Fujiwara Y., Masutani C., Mizukoshi T., Kondo J., Hanaoka F., Iwai S. (1999). Characterization of DNA recognition by the human UV-damaged DNA-binding protein. J. Biol. Chem..

[24] Groisman R., Polanowska J., Kuraoka I., Sawada J., Saijo M., Drapkin R., Kisselev A.F., Tanaka K., Nakatani Y. (2003). The ubiquitin ligase activity in the DDB2 and CSA complexes is differentially regulated by the COP9 signalosome in response to DNA damage. Cell.

[25] Fischer E.S., Scrima A., Bohm K., Matsumoto S., Lingaraju G.M., Faty M., Yasuda T., Cavadini S., Wakasugi M., Hanaoka F. (2011). The molecular basis of CRL4DDB2/CSA ubiquitin ligase architecture, targeting, and activation. Cell.

[26] Tang J.Y., Hwang B.J., Ford J.M., Hanawalt P.C., Chu G. (2000). Xeroderma pigmentosum gene enhances global genomic repair and suppresses UV-induced mutagenesis. Mol. Cell.

[27] Slyskova J., Muniesa-Vargas A., da Silva I.T., Drummond R., Park J., Hackes D., Poetsch I., Ribeiro-Silva C., Moretton A., Heffeter P. (2023). Detection of oxaliplatin- and cisplatin-DNA lesions requires different global genome repair mechanisms that affect their clinical efficacy. NAR Cancer.

[28] Li C.L., Golebiowski F.M., Onishi Y., Samara N.L., Sugasawa K., Yang W. (2015). Tripartite DNA Lesion Recognition and Verification by XPC, TFIIH, and XPA in Nucleotide Excision Repair. Mol. Cell.

[29] Coin F., Oksenych V., Mocquet V., Groh S., Blattner C., Egly J.M. (2008). Nucleotide excision repair driven by the dissociation of CAK from TFIIH. Mol. Cell.

[30] Kokic G., Chernev A., Tegunov D., Dienemann C., Urlaub H., Cramer P. (2019). Structural basis of TFIIH activation for nucleotide excision repair. Nat. Commun..

[31] Bralic A., Tehseen M., Sobhy M.A., Tsai C.L., Alhudhali L., Yi G., Yu J., Yan C., Ivanov I., Tsutakawa S.E. (2023). A scanning-to-incision switch in TFIIH-XPG induced by DNA damage licenses nucleotide excision repair. Nucleic Acids Res..

[32] Naegeli H., Modrich P., Friedberg E.C. (1993). The DNA helicase activities of Rad3 protein of Saccharomyces cerevisiae and helicase II of Escherichia coli are differentially inhibited by covalent and noncovalent DNA modifications. J. Biol. Chem..

[33] Coin F., Oksenych V., Egly J.M. (2007). Distinct roles for the XPB/p52 and XPD/p44 subcomplexes of TFIIH in damaged DNA opening during nucleotide excision repair. Mol. Cell.

[34] Mathieu N., Kaczmarek N., Naegeli H. (2010). Strand- and site-specific DNA lesion demarcation by the xeroderma pigmentosum group D helicase. Proc. Natl. Acad. Sci. USA.

[35] Buechner C.N., Heil K., Michels G., Carell T., Kisker C., Tessmer I. (2014). Strand-specific recognition of DNA damages by XPD provides insights into nucleotide excision repair substrate versatility. J. Biol. Chem..

[36] Sugasawa K., Akagi J., Nishi R., Iwai S., Hanaoka F. (2009). Two-step recognition of DNA damage for mammalian nucleotide excision repair: Directional binding of the XPC complex and DNA strand scanning. Mol. Cell.

[37] Yokoi M., Masutani C., Maekawa T., Sugasawa K., Ohkuma Y., Hanaoka F. (2000). The xeroderma pigmentosum group C protein complex XPC-HR23B plays an important role in the recruitment of transcription factor IIH to damaged DNA. J. Biol. Chem..

[38] Oksenych V., de Jesus B.B., Zhovmer A., Egly J.M., Coin F. (2009). Molecular insights into the recruitment of TFIIH to sites of DNA damage. EMBO J..

[39] van Eeuwen T., Shim Y., Kim H.J., Zhao T., Basu S., Garcia B.A., Kaplan C.D., Min J.H., Murakami K. (2021). Cryo-EM structure of TFIIH/Rad4-Rad23-Rad33 in damaged DNA opening in nucleotide excision repair. Nat. Commun..

[40] Coin F., Proietti De Santis L., Nardo T., Zlobinskaya O., Stefanini M., Egly J.M. (2006). p8/TTD-A as a repair-specific TFIIH subunit. Mol. Cell.

[41] Fu I., Mu H., Geacintov N.E., Broyde S. (2022). Mechanism of lesion verification by the human XPD helicase in nucleotide excision repair. Nucleic Acids Res..

[42] Fu I., Geacintov N.E., Broyde S. (2023). Differing structures and dynamics of two photolesions portray verification differences by the human XPD helicase. Nucleic Acids Res..

[43] Kuper J., Hove T., Maidl S., Neitz H., Sauer F., Kempf M., Schroeder T., Greiter E., Hobartner C., Kisker C. (2024). XPD stalled on cross-linked DNA provides insight into damage verification. Nat. Struct. Mol. Biol..

[44] Wirth N., Gross J., Roth H.M., Buechner C.N., Kisker C., Tessmer I. (2016). Conservation and Divergence in Nucleotide Excision Repair Lesion Recognition. J. Biol. Chem..

[45] Fagbemi A.F., Orelli B., Scharer O.D. (2011). Regulation of endonuclease activity in human nucleotide excision repair. DNA Repair.

[46] Yu J., Yan C., Paul T., Brewer L., Tsutakawa S.E., Tsai C.L., Hamdan S.M., Tainer J.A., Ivanov I. (2024). Molecular architecture and functional dynamics of the pre-incision complex in nucleotide excision repair. Nat. Commun..

[47] Sugasawa K. (2016). Molecular mechanisms of DNA damage recognition for mammalian nucleotide excision repair. DNA Repair.

[48] Gsell C., Richly H., Coin F., Naegeli H. (2020). A chromatin scaffold for DNA damage recognition: How histone methyltransferases prime nucleosomes for repair of ultraviolet light-induced lesions. Nucleic Acids Res..

[49] Tsutakawa S.E., Tsai C.L., Yan C., Bralic A., Chazin W.J., Hamdan S.M., Scharer O.D., Ivanov I., Tainer J.A. (2020). Envisioning how the prototypic molecular machine TFIIH functions in transcription initiation and DNA repair. DNA Repair.

[50] Theil A.F., Hackes D., Lans H. (2023). TFIIH central activity in nucleotide excision repair to prevent disease. DNA Repair.

[51] Wittschieben B.O., Iwai S., Wood R.D. (2005). DDB1-DDB2 (xeroderma pigmentosum group E) protein complex recognizes a cyclobutane pyrimidine dimer, mismatches, apurinic/apyrimidinic sites, and compound lesions in DNA. J. Biol. Chem..

[52] Hanawalt P.C., Spivak G. (2008). Transcription-coupled DNA repair: Two decades of progress and surprises. Nat. Rev. Mol. Cell Biol..

[53] Jia N., Guo C., Nakazawa Y., van den Heuvel D., Luijsterburg M.S., Ogi T. (2021). Dealing with transcription-blocking DNA damage: Repair mechanisms, RNA polymerase II processing and human disorders. DNA Repair.

[54] van den Heuvel D., van der Weegen Y., Boer D.E.C., Ogi T., Luijsterburg M.S. (2021). Transcription-Coupled DNA Repair: From Mechanism to Human Disorder. Trends Cell Biol..

[55] Selby C.P., Lindsey-Boltz L.A., Li W., Sancar A. (2023). Molecular Mechanisms of Transcription-Coupled Repair. Annu. Rev. Biochem..

[56] Brueckner F., Hennecke U., Carell T., Cramer P. (2007). CPD damage recognition by transcribing RNA polymerase II. Science.

[57] Damsma G.E., Alt A., Brueckner F., Carell T., Cramer P. (2007). Mechanism of transcriptional stalling at cisplatin-damaged DNA. Nat. Struct. Mol. Biol..

[58] Selby C.P., Sancar A. (1997). Cockayne syndrome group B protein enhances elongation by RNA polymerase II. Proc. Natl. Acad. Sci. USA.

[59] Cho I., Tsai P.F., Lake R.J., Basheer A., Fan H.Y. (2013). ATP-dependent chromatin remodeling by Cockayne syndrome protein B and NAP1-like histone chaperones is required for efficient transcription-coupled DNA repair. PLoS Genet..

[60] Xu J., Wang W., Xu L., Chen J.Y., Chong J., Oh J., Leschziner A.E., Fu X.D., Wang D. (2020). Cockayne syndrome B protein acts as an ATP-dependent processivity factor that helps RNA polymerase II overcome nucleosome barriers. Proc. Natl. Acad. Sci. USA.

[61] van Gool A.J., Citterio E., Rademakers S., van Os R., Vermeulen W., Constantinou A., Egly J.M., Bootsma D., Hoeijmakers J.H. (1997). The Cockayne syndrome B protein, involved in transcription-coupled DNA repair, resides in an RNA polymerase II-containing complex. EMBO J..

[62] van den Boom V., Citterio E., Hoogstraten D., Zotter A., Egly J.M., van Cappellen W.A., Hoeijmakers J.H., Houtsmuller A.B., Vermeulen W. (2004). DNA damage stabilizes interaction of CSB with the transcription elongation machinery. J. Cell Biol..

[63] Charlet-Berguerand N., Feuerhahn S., Kong S.E., Ziserman H., Conaway J.W., Conaway R., Egly J.M. (2006). RNA polymerase II bypass of oxidative DNA damage is regulated by transcription elongation factors. EMBO J..

[64] Kokic G., Wagner F.R., Chernev A., Urlaub H., Cramer P. (2021). Structural basis of human transcription-DNA repair coupling. Nature.

[65] Xu J., Lahiri I., Wang W., Wier A., Cianfrocco M.A., Chong J., Hare A.A., Dervan P.B., DiMaio F., Leschziner A.E. (2017). Structural basis for the initiation of eukaryotic transcription-coupled DNA repair. Nature.

[66] van der Weegen Y., Golan-Berman H., Mevissen T.E.T., Apelt K., Gonzalez-Prieto R., Goedhart J., Heilbrun E.E., Vertegaal A.C.O., van den Heuvel D., Walter J.C. (2020). The cooperative action of CSB, CSA, and UVSSA target TFIIH to DNA damage-stalled RNA polymerase II. Nat. Commun..

[67] Venema J., Mullenders L.H., Natarajan A.T., van Zeeland A.A., Mayne L.V. (1990). The genetic defect in Cockayne syndrome is associated with a defect in repair of UV-induced DNA damage in transcriptionally active DNA. Proc. Natl. Acad. Sci. USA.

[68] Nakazawa Y., Hara Y., Oka Y., Komine O., van den Heuvel D., Guo C., Daigaku Y., Isono M., He Y., Shimada M. (2020). Ubiquitination of DNA Damage-Stalled RNAPII Promotes Transcription-Coupled Repair. Cell.

[69] Tufegdzic Vidakovic A., Mitter R., Kelly G.P., Neumann M., Harreman M., Rodriguez-Martinez M., Herlihy A., Weems J.C., Boeing S., Encheva V. (2020). Regulation of the RNAPII Pool Is Integral to the DNA Damage Response. Cell.

[70] Fei J., Chen J. (2012). KIAA1530 protein is recruited by Cockayne syndrome complementation group protein A (CSA) to participate in transcription-coupled repair (TCR). J. Biol. Chem..

[71] Nakazawa Y., Sasaki K., Mitsutake N., Matsuse M., Shimada M., Nardo T., Takahashi Y., Ohyama K., Ito K., Mishima H. (2012). Mutations in UVSSA cause UV-sensitive syndrome and impair RNA polymerase IIo processing in transcription-coupled nucleotide-excision repair. Nat. Genet..

[72] Schwertman P., Lagarou A., Dekkers D.H., Raams A., van der Hoek A.C., Laffeber C., Hoeijmakers J.H., Demmers J.A., Fousteri M., Vermeulen W. (2012). UV-sensitive syndrome protein UVSSA recruits USP7 to regulate transcription-coupled repair. Nat. Genet..

[73] Zhang X., Horibata K., Saijo M., Ishigami C., Ukai A., Kanno S., Tahara H., Neilan E.G., Honma M., Nohmi T. (2012). Mutations in UVSSA cause UV-sensitive syndrome and destabilize ERCC6 in transcription-coupled DNA repair. Nat. Genet..

[74] Okuda M., Nakazawa Y., Guo C., Ogi T., Nishimura Y. (2017). Common TFIIH recruitment mechanism in global genome and transcription-coupled repair subpathways. Nucleic Acids Res..

[75] Olivieri M., Cho T., Alvarez-Quilon A., Li K., Schellenberg M.J., Zimmermann M., Hustedt N., Rossi S.E., Adam S., Melo H. (2020). A Genetic Map of the Response to DNA Damage in Human Cells. Cell.

[76] van der Weegen Y., de Lint K., van den Heuvel D., Nakazawa Y., Mevissen T.E.T., van Schie J.J.M., San Martin Alonso M., Boer D.E.C., Gonzalez-Prieto R., Narayanan I.V. (2021). ELOF1 is a transcription-coupled DNA repair factor that directs RNA polymerase II ubiquitylation. Nat. Cell Biol..

[77] Geijer M.E., Zhou D., Selvam K., Steurer B., Mukherjee C., Evers B., Cugusi S., van Toorn M., van der Woude M., Janssens R.C. (2021). Elongation factor ELOF1 drives transcription-coupled repair and prevents genome instability. Nat. Cell Biol..

[78] Mevissen T.E.T., Kummecke M., Schmid E.W., Farnung L., Walter J.C. (2024). STK19 positions TFIIH for cell-free transcription-coupled DNA repair. Cell.

[79] Ramadhin A.R., Lee S.H., Zhou D., Salmazo A., Gonzalo-Hansen C., van Sluis M., Blom C.M.A., Janssens R.C., Raams A., Dekkers D. (2024). STK19 drives transcription-coupled repair by stimulating repair complex stability, RNA Pol II ubiquitylation, and TFIIH recruitment. Mol. Cell.

[80] Tan Y., Gao M., Huang Y., Zhan D., Wu S., An J., Zhang X., Hu J. (2024). STK19 is a transcription-coupled repair factor that participates in UVSSA ubiquitination and TFIIH loading. Nucleic Acids Res..

[81] van den Heuvel D., Rodriguez-Martinez M., van der Meer P.J., Nieto Moreno N., Park J., Kim H.S., van Schie J.J.M., Wondergem A.P., D’Souza A., Yakoub G. (2024). STK19 facilitates the clearance of lesion-stalled RNAPII during transcription-coupled DNA repair. Cell.

[82] Nakatsu Y., Asahina H., Citterio E., Rademakers S., Vermeulen W., Kamiuchi S., Yeo J.P., Khaw M.C., Saijo M., Kodo N. (2000). XAB2, a novel tetratricopeptide repeat protein involved in transcription-coupled DNA repair and transcription. J. Biol. Chem..

[83] Zhu Y., Zhang X., Gao M., Huang Y., Tan Y., Parnas A., Wu S., Zhan D., Adar S., Hu J. (2024). Coordination of transcription-coupled repair and repair-independent release of lesion-stalled RNA polymerase II. Nat. Commun..

[84] Paul T., Yan C., Yu J., Tsutakawa S.E., Tainer J.A., Wang D., Ivanov I. (2025). Molecular model of TFIIH recruitment to the transcription-coupled repair machinery. Nat. Commun..

[85] Cleaver J.E. (1968). Defective Repair Replication of DNA in Xeroderma Pigmentosum. Nature.

[86] Limsirichaikul S., Niimi A., Fawcett H., Lehmann A., Yamashita S., Ogi T. (2009). A rapid non-radioactive technique for measurement of repair synthesis in primary human fibroblasts by incorporation of ethynyl deoxyuridine (EdU). Nucleic Acids Res..

[87] Pimpley M.R., Foley M.L., Latimer J.J. (2020). New Perspectives on Unscheduled DNA Synthesis: Functional Assay for Global Genomic DNA Nucleotide Excision Repair. Methods Mol. Biol..

[88] van der Meer P.J., Van Den Heuvel D., Luijsterburg M.S. (2023). Unscheduled DNA Synthesis at Sites of Local UV-induced DNA Damage to Quantify Global Genome Nucleotide Excision Repair Activity in Human Cells. Bio Protoc..

[89] Wienholz F., Vermeulen W., Marteijn J.A. (2017). Amplification of unscheduled DNA synthesis signal enables fluorescence-based single cell quantification of transcription-coupled nucleotide excision repair. Nucleic Acids Res..

[90] van der Woude M., Davo-Martinez C., Thijssen K.L., Vermeulen W., Lans H. (2023). Recovery of protein synthesis to assay DNA repair activity in transcribed genes in living cells and tissues. Nucleic Acids Res..

[91] van Hoffen A., Venema J., Meschini R., van Zeeland A.A., Mullenders L.H. (1995). Transcription-coupled repair removes both cyclobutane pyrimidine dimers and 6-4 photoproducts with equal efficiency and in a sequential way from transcribed DNA in xeroderma pigmentosum group C fibroblasts. EMBO J..

[92] Mayne L.V., Lehmann A.R. (1982). Failure of RNA synthesis to recover after UV irradiation: An early defect in cells from individuals with Cockayne’s syndrome and xeroderma pigmentosum. Cancer Res..

[93] Lehmann A.R., Francis A.J., Giannelli F. (1985). Prenatal-Diagnosis of Cockaynes Syndrome. Lancet.

[94] Lehmann A.R., Thompson A.F., Harcourt S.A., Stefanini M., Norris P.G. (1993). Cockayne’s syndrome: Correlation of clinical features with cellular sensitivity of RNA synthesis to UV irradiation. J. Med. Genet..

[95] Nakazawa Y., Yamashita S., Lehmann A.R., Ogi T. (2010). A semi-automated non-radioactive system for measuring recovery of RNA synthesis and unscheduled DNA synthesis using ethynyluracil derivatives. DNA Repair.

[96] Geijer M.E., Marteijn J.A. (2018). What happens at the lesion does not stay at the lesion: Transcription-coupled nucleotide excision repair and the effects of DNA damage on transcription in cis and trans. DNA Repair.

[97] Khobta A., Epe B. (2012). Interactions between DNA damage, repair, and transcription. Mutat. Res..

[98] Hu J., Adar S., Selby C.P., Lieb J.D., Sancar A. (2015). Genome-wide analysis of human global and transcription-coupled excision repair of UV damage at single-nucleotide resolution. Genes. Dev..

[99] Adar S., Hu J., Lieb J.D., Sancar A. (2016). Genome-wide kinetics of DNA excision repair in relation to chromatin state and mutagenesis. Proc. Natl. Acad. Sci. USA.

[100] Kemp M.G., Reardon J.T., Lindsey-Boltz L.A., Sancar A. (2012). Mechanism of release and fate of excised oligonucleotides during nucleotide excision repair. J. Biol. Chem..

[101] Hu J., Li W., Adebali O., Yang Y., Oztas O., Selby C.P., Sancar A. (2019). Genome-wide mapping of nucleotide excision repair with XR-seq. Nat. Protoc..

[102] Li W., Hu J., Adebali O., Adar S., Yang Y., Chiou Y.Y., Sancar A. (2017). Human genome-wide repair map of DNA damage caused by the cigarette smoke carcinogen benzo[a]pyrene. Proc. Natl. Acad. Sci. USA.

[103] Hu J., Lieb J.D., Sancar A., Adar S. (2016). Cisplatin DNA damage and repair maps of the human genome at single-nucleotide resolution. Proc. Natl. Acad. Sci. USA.

[104] Zhu Y., Tan Y., Li L., Xiang Y., Huang Y., Zhang X., Yin J., Li J., Lan F., Qian M. (2023). Genome-wide mapping of protein-DNA damage interaction by PADD-seq. Nucleic Acids Res..

[105] Mao P., Smerdon M.J., Roberts S.A., Wyrick J.J. (2016). Chromosomal landscape of UV damage formation and repair at single-nucleotide resolution. Proc. Natl. Acad. Sci. USA.

[106] Jiang Y., Li W., Lindsey-Boltz L.A., Yang Y., Li Y., Sancar A. (2021). Super hotspots and super coldspots in the repair of UV-induced DNA damage in the human genome. J. Biol. Chem..

[107] Wu Y., Adeel M.M., Xia D., Sancar A., Li W. (2024). Nucleotide excision repair of aflatoxin-induced DNA damage within the 3D human genome organization. Nucleic Acids Res..

[108] Haradhvala N.J., Polak P., Stojanov P., Covington K.R., Shinbrot E., Hess J.M., Rheinbay E., Kim J., Maruvka Y.E., Braunstein L.Z. (2016). Mutational Strand Asymmetries in Cancer Genomes Reveal Mechanisms of DNA Damage and Repair. Cell.

[109] Alexandrov L.B., Kim J., Haradhvala N.J., Huang M.N., Tian Ng A.W., Wu Y., Boot A., Covington K.R., Gordenin D.A., Bergstrom E.N. (2020). The repertoire of mutational signatures in human cancer. Nature.

[110] Pleasance E.D., Cheetham R.K., Stephens P.J., McBride D.J., Humphray S.J., Greenman C.D., Varela I., Lin M.L., Ordonez G.R., Bignell G.R. (2010). A comprehensive catalogue of somatic mutations from a human cancer genome. Nature.

[111] Laine J.P., Egly J.M. (2006). Initiation of DNA repair mediated by a stalled RNA polymerase IIO. EMBO J..

[112] Araujo S.J., Tirode F., Coin F., Pospiech H., Syvaoja J.E., Stucki M., Hubscher U., Egly J.M., Wood R.D. (2000). Nucleotide excision repair of DNA with recombinant human proteins: Definition of the minimal set of factors, active forms of TFIIH, and modulation by CAK. Genes. Dev..

[113] Donahue B.A., Yin S., Taylor J.S., Reines D., Hanawalt P.C. (1994). Transcript cleavage by RNA polymerase II arrested by a cyclobutane pyrimidine dimer in the DNA template. Proc. Natl. Acad. Sci. USA.

[114] Tornaletti S., Reines D., Hanawalt P.C. (1999). Structural characterization of RNA polymerase II complexes arrested by a cyclobutane pyrimidine dimer in the transcribed strand of template DNA. J. Biol. Chem..

[115] Tornaletti S., Patrick S.M., Turchi J.J., Hanawalt P.C. (2003). Behavior of T7 RNA polymerase and mammalian RNA polymerase II at site-specific cisplatin adducts in the template DNA. J. Biol. Chem..

[116] Tornaletti S., Hanawalt P.C. (1999). Effect of DNA lesions on transcription elongation. Biochimie.

[117] Wang W., Xu J., Chong J., Wang D. (2018). Structural basis of DNA lesion recognition for eukaryotic transcription-coupled nucleotide excision repair. DNA Repair.

[118] Agapov A., Olina A., Kulbachinskiy A. (2022). RNA polymerase pausing, stalling and bypass during transcription of damaged DNA: From molecular basis to functional consequences. Nucleic Acids Res..

[119] Cheng T.F., Hu X., Gnatt A., Brooks P.J. (2008). Differential blocking effects of the acetaldehyde-derived DNA lesion N2-ethyl-2’-deoxyguanosine on transcription by multisubunit and single subunit RNA polymerases. J. Biol. Chem..

[120] Dimitri A., Goodenough A.K., Guengerich F.P., Broyde S., Scicchitano D.A. (2008). Transcription processing at 1,N2-ethenoguanine by human RNA polymerase II and bacteriophage T7 RNA polymerase. J. Mol. Biol..

[121] Walmacq C., Wang L., Chong J., Scibelli K., Lubkowska L., Gnatt A., Brooks P.J., Wang D., Kashlev M. (2015). Mechanism of RNA polymerase II bypass of oxidative cyclopurine DNA lesions. Proc. Natl. Acad. Sci. USA.

[122] Oh J., Fleming A.M., Xu J., Chong J., Burrows C.J., Wang D. (2020). RNA polymerase II stalls on oxidative DNA damage via a torsion-latch mechanism involving lone pair-pi and CH-pi interactions. Proc. Natl. Acad. Sci. USA.

[123] Walmacq C., Cheung A.C., Kireeva M.L., Lubkowska L., Ye C., Gotte D., Strathern J.N., Carell T., Cramer P., Kashlev M. (2012). Mechanism of translesion transcription by RNA polymerase II and its role in cellular resistance to DNA damage. Mol. Cell.

[124] Li W., Selvam K., Ko T., Li S. (2014). Transcription bypass of DNA lesions enhances cell survival but attenuates transcription coupled DNA repair. Nucleic Acids Res..

[125] Alanazi J.S., Latimer J.J. (2020). Host Cell Reactivation: Assay for Actively Transcribed DNA (Nucleotide Excision) Repair Using Luciferase Family Expression Vectors. Methods Mol. Biol..

[126] Protic-Sabljic M., Kraemer K.H. (1985). One pyrimidine dimer inactivates expression of a transfected gene in xeroderma pigmentosum cells. Proc. Natl. Acad. Sci. USA.

[127] Lehmann A.R., Oomen A. (1985). Effect of DNA damage on the expression of the chloramphenicol acetyltransferase gene after transfection into diploid human fibroblasts. Nucleic Acids Res..

[128] Abrahams P.J., Van der Eb A.J. (1976). Host-cell reactivation of ultraviolet-irradiated SV40 DNA in five complementation groups of xeroderma pigmentosum. Mutat. Res..

[129] Selsky C.A., Greer S. (1978). Host-cell reactivation of UV-irradiated and chemically-treated herpes simplex virus-1 by xeroderma pigmentosum, XP heterozygotes and normal skin fibroblasts. Mutat. Res..

[130] Barrett S.F., Robbins J.H., Tarone R.E., Kraemer K.H. (1991). Evidence for defective repair of cyclobutane pyrimidine dimers with normal repair of other DNA photoproducts in a transcriptionally active gene transfected into Cockayne syndrome cells. Mutat. Res..

[131] Cordeiro-Stone M., Zaritskaya L.S., Price L.K., Kaufmann W.K. (1997). Replication fork bypass of a pyrimidine dimer blocking leading strand DNA synthesis. J. Biol. Chem..

[132] Wang H., Hays J.B. (2001). Simple and rapid preparation of gapped plasmid DNA for incorporation of oligomers containing specific DNA lesions. Mol. Biotechnol..

[133] Baker D.J., Wuenschell G., Xia L., Termini J., Bates S.E., Riggs A.D., O’Connor T.R. (2007). Nucleotide excision repair eliminates unique DNA-protein cross-links from mammalian cells. J. Biol. Chem..

[134] Burns J.A., Dreij K., Cartularo L., Scicchitano D.A. (2010). O6-methylguanine induces altered proteins at the level of transcription in human cells. Nucleic Acids Res..

[135] Enoiu M., Jiricny J., Scharer O.D. (2012). Repair of cisplatin-induced DNA interstrand crosslinks by a replication-independent pathway involving transcription-coupled repair and translesion synthesis. Nucleic Acids Res..

[136] Luhnsdorf B., Kitsera N., Warken D., Lingg T., Epe B., Khobta A. (2012). Generation of reporter plasmids containing defined base modifications in the DNA strand of choice. Anal. Biochem..

[137] You C., Wang Y. (2015). Quantitative measurement of transcriptional inhibition and mutagenesis induced by site-specifically incorporated DNA lesions in vitro and in vivo. Nat. Protoc..

[138] Piett C.G., Pecen T.J., Laverty D.J., Nagel Z.D. (2021). Large-scale preparation of fluorescence multiplex host cell reactivation (FM-HCR) reporters. Nat. Protoc..

[139] Kitsera N., Rodriguez-Alvarez M., Emmert S., Carell T., Khobta A. (2019). Nucleotide excision repair of abasic DNA lesions. Nucleic Acids Res..

[140] Sarmini L., Meabed M., Emmanouil E., Atsaves G., Robeska E., Karwowski B.T., Campalans A., Gimisis T., Khobta A. (2023). Requirement of transcription-coupled nucleotide excision repair for the removal of a specific type of oxidatively induced DNA damage. Nucleic Acids Res..

[141] You C., Dai X., Yuan B., Wang J., Wang J., Brooks P.J., Niedernhofer L.J., Wang Y. (2012). A quantitative assay for assessing the effects of DNA lesions on transcription. Nat. Chem. Biol..

[142] Kitsera N., Gasteiger K., Luhnsdorf B., Allgayer J., Epe B., Carell T., Khobta A. (2014). Cockayne syndrome: Varied requirement of transcription-coupled nucleotide excision repair for the removal of three structurally different adducts from transcribed DNA. PLoS ONE.

[143] Nagel Z.D., Margulies C.M., Chaim I.A., McRee S.K., Mazzucato P., Ahmad A., Abo R.P., Butty V.L., Forget A.L., Samson L.D. (2014). Multiplexed DNA repair assays for multiple lesions and multiple doses via transcription inhibition and transcriptional mutagenesis. Proc. Natl. Acad. Sci. USA.

[144] Chaim I.A., Gardner A., Wu J., Iyama T., Wilson D.M., Samson L.D. (2017). A novel role for transcription-coupled nucleotide excision repair for the in vivo repair of 3,N4-ethenocytosine. Nucleic Acids Res..

[145] Sarmini L., Kitsera N., Meabed M., Khobta A. (2025). Transcription blocking properties and transcription-coupled repair of N(2)-alkylguanine adducts as a model for aldehyde-induced DNA damage. J. Biol. Chem..

[146] You C., Wang J., Dai X., Wang Y. (2015). Transcriptional inhibition and mutagenesis induced by N-nitroso compound-derived carboxymethylated thymidine adducts in DNA. Nucleic Acids Res..

[147] Mouret S., Baudouin C., Charveron M., Favier A., Cadet J., Douki T. (2006). Cyclobutane pyrimidine dimers are predominant DNA lesions in whole human skin exposed to UVA radiation. Proc. Natl. Acad. Sci. USA.

[148] Mei Kwei J.S., Kuraoka I., Horibata K., Ubukata M., Kobatake E., Iwai S., Handa H., Tanaka K. (2004). Blockage of RNA polymerase II at a cyclobutane pyrimidine dimer and 6-4 photoproduct. Biochem. Biophys. Res. Commun..

[149] Reardon J.T., Sancar A. (2003). Recognition and repair of the cyclobutane thymine dimer, a major cause of skin cancers, by the human excision nuclease. Genes. Dev..

[150] Laughery M.F., Brown A.J., Bohm K.A., Sivapragasam S., Morris H.S., Tchmola M., Washington A.D., Mitchell D., Mather S., Malc E.P. (2020). Atypical UV Photoproducts Induce Non-canonical Mutation Classes Associated with Driver Mutations in Melanoma. Cell Rep..

[151] Hess M.T., Gunz D., Luneva N., Geacintov N.E., Naegeli H. (1997). Base pair conformation-dependent excision of benzo[a]pyrene diol epoxide-guanine adducts by human nucleotide excision repair enzymes. Mol. Cell Biol..

[152] Buterin T., Hess M.T., Luneva N., Geacintov N.E., Amin S., Kroth H., Seidel A., Naegeli H. (2000). Unrepaired fjord region polycyclic aromatic hydrocarbon-DNA adducts in ras codon 61 mutational hot spots. Cancer Res..

[153] Kusumoto R., Masutani C., Sugasawa K., Iwai S., Araki M., Uchida A., Mizukoshi T., Hanaoka F. (2001). Diversity of the damage recognition step in the global genomic nucleotide excision repair in vitro. Mutat. Res..

[154] Mocquet V., Kropachev K., Kolbanovskiy M., Kolbanovskiy A., Tapias A., Cai Y., Broyde S., Geacintov N.E., Egly J.M. (2007). The human DNA repair factor XPC-HR23B distinguishes stereoisomeric benzo[a]pyrenyl-DNA lesions. EMBO J..

[155] Mu H., Kropachev K., Wang L., Zhang L., Kolbanovskiy A., Kolbanovskiy M., Geacintov N.E., Broyde S. (2012). Nucleotide excision repair of 2-acetylaminofluorene- and 2-aminofluorene-(C8)-guanine adducts: Molecular dynamics simulations elucidate how lesion structure and base sequence context impact repair efficiencies. Nucleic Acids Res..

[156] Kropachev K., Kolbanovskiy M., Liu Z., Cai Y., Zhang L., Schwaid A.G., Kolbanovskiy A., Ding S., Amin S., Broyde S. (2013). Adenine-DNA adducts derived from the highly tumorigenic Dibenzo[a,l]pyrene are resistant to nucleotide excision repair while guanine adducts are not. Chem. Res. Toxicol..

[157] Nadkarni A., Burns J.A., Gandolfi A., Chowdhury M.A., Cartularo L., Berens C., Geacintov N.E., Scicchitano D.A. (2016). Nucleotide Excision Repair and Transcription-coupled DNA Repair Abrogate the Impact of DNA Damage on Transcription. J. Biol. Chem..

[158] Mu H., Geacintov N.E., Min J.H., Zhang Y., Broyde S. (2017). Nucleotide Excision Repair Lesion-Recognition Protein Rad4 Captures a Pre-Flipped Partner Base in a Benzo[a]pyrene-Derived DNA Lesion: How Structure Impacts the Binding Pathway. Chem. Res. Toxicol..

[159] Olivier M., Weninger A., Ardin M., Huskova H., Castells X., Vallee M.P., McKay J., Nedelko T., Muehlbauer K.R., Marusawa H. (2014). Modelling mutational landscapes of human cancers in vitro. Sci. Rep..

[160] Severson P.L., Vrba L., Stampfer M.R., Futscher B.W. (2014). Exome-wide mutation profile in benzo[a]pyrene-derived post-stasis and immortal human mammary epithelial cells. Mutat. Res. Genet. Toxicol. Environ. Mutagen..

[161] Nik-Zainal S., Kucab J.E., Morganella S., Glodzik D., Alexandrov L.B., Arlt V.M., Weninger A., Hollstein M., Stratton M.R., Phillips D.H. (2015). The genome as a record of environmental exposure. Mutagenesis.

[162] Kucab J.E., Zou X., Morganella S., Joel M., Nanda A.S., Nagy E., Gomez C., Degasperi A., Harris R., Jackson S.P. (2019). A Compendium of Mutational Signatures of Environmental Agents. Cell.

[163] Mingard C., Battey J.N.D., Takhaveev V., Blatter K., Hurlimann V., Sierro N., Ivanov N.V., Sturla S.J. (2023). Dissection of Cancer Mutational Signatures with Individual Components of Cigarette Smoking. Chem. Res. Toxicol..

[164] Heflich R.H., Neft R.E. (1994). Genetic toxicity of 2-acetylaminofluorene, 2-aminofluorene and some of their metabolites and model metabolites. Mutat. Res..

[165] O’Handley S.F., Sanford D.G., Xu R., Lester C.C., Hingerty B.E., Broyde S., Krugh T.R. (1993). Structural characterization of an N-acetyl-2-aminofluorene (AAF) modified DNA oligomer by NMR, energy minimization, and molecular dynamics. Biochemistry.

[166] Zaliznyak T., Bonala R., Johnson F., de Los Santos C. (2006). Structure and stability of duplex DNA containing the 3-(deoxyguanosin-N2-yl)-2-acetylaminofluorene (dG(N2)-AAF) lesion: A bulky adduct that persists in cellular DNA. Chem. Res. Toxicol..

[167] Cui X.S., Eriksson L.C., Moller L. (1999). Formation and persistence of DNA adducts during and after a long-term administration of 2-nitrofluorene. Mutat. Res..

[168] Turesky R.J., Markovic J., Aeschlimann J.M. (1996). Formation and differential removal of C-8 and N2-guanine adducts of the food carcinogen 2-amino-3-methylimidazo[4,5-f]quinoline in the liver, kidney, and colorectum of the rat. Chem. Res. Toxicol..

[169] Pfau W., Schmeiser H.H., Wiessler M. (1990). Aristolochic acid binds covalently to the exocyclic amino group of purine nucleotides in DNA. Carcinogenesis.

[170] Stiborova M., Frei E., Sopko B., Sopkova K., Markova V., Lankova M., Kumstyrova T., Wiessler M., Schmeiser H.H. (2003). Human cytosolic enzymes involved in the metabolic activation of carcinogenic aristolochic acid: Evidence for reductive activation by human NAD(P)H:quinone oxidoreductase. Carcinogenesis.

[171] Sidorenko V.S., Yeo J.E., Bonala R.R., Johnson F., Scharer O.D., Grollman A.P. (2012). Lack of recognition by global-genome nucleotide excision repair accounts for the high mutagenicity and persistence of aristolactam-DNA adducts. Nucleic Acids Res..

[172] Chen C.H., Dickman K.G., Moriya M., Zavadil J., Sidorenko V.S., Edwards K.L., Gnatenko D.V., Wu L., Turesky R.J., Wu X.R. (2012). Aristolochic acid-associated urothelial cancer in Taiwan. Proc. Natl. Acad. Sci. USA.

[173] Hoang M.L., Chen C.H., Sidorenko V.S., He J., Dickman K.G., Yun B.H., Moriya M., Niknafs N., Douville C., Karchin R. (2013). Mutational signature of aristolochic acid exposure as revealed by whole-exome sequencing. Sci. Transl. Med..

[174] Poon S.L., Huang M.N., Choo Y., McPherson J.R., Yu W., Heng H.L., Gan A., Myint S.S., Siew E.Y., Ler L.D. (2015). Mutation signatures implicate aristolochic acid in bladder cancer development. Genome Med..

[175] Groopman J.D., Croy R.G., Wogan G.N. (1981). In vitro reactions of aflatoxin B1-adducted DNA. Proc. Natl. Acad. Sci. USA.

[176] Chawanthayatham S., Valentine C.C., Fedeles B.I., Fox E.J., Loeb L.A., Levine S.S., Slocum S.L., Wogan G.N., Croy R.G., Essigmann J.M. (2017). Mutational spectra of aflatoxin B(1) in vivo establish biomarkers of exposure for human hepatocellular carcinoma. Proc. Natl. Acad. Sci. USA.

[177] Jamieson E.R., Lippard S.J. (1999). Structure, Recognition, and Processing of Cisplatin-DNA Adducts. Chem. Rev..

[178] Huang J.C., Zamble D.B., Reardon J.T., Lippard S.J., Sancar A. (1994). HMG-domain proteins specifically inhibit the repair of the major DNA adduct of the anticancer drug cisplatin by human excision nuclease. Proc. Natl. Acad. Sci. USA.

[179] Boot A., Huang M.N., Ng A.W.T., Ho S.C., Lim J.Q., Kawakami Y., Chayama K., Teh B.T., Nakagawa H., Rozen S.G. (2018). In-depth characterization of the cisplatin mutational signature in human cell lines and in esophageal and liver tumors. Genome Res..

[180] Neels J.F., Gong J., Yu X., Sturla S.J. (2007). Quantitative correlation of drug bioactivation and deoxyadenosine alkylation by acylfulvene. Chem. Res. Toxicol..

[181] Pietsch K.E., van Midwoud P.M., Villalta P.W., Sturla S.J. (2013). Quantification of acylfulvene- and illudin S-DNA adducts in cells with variable bioactivation capacities. Chem. Res. Toxicol..

[182] Gong J., Vaidyanathan V.G., Yu X., Kensler T.W., Peterson L.A., Sturla S.J. (2007). Depurinating acylfulvene-DNA adducts: Characterizing cellular chemical reactions of a selective antitumor agent. J. Am. Chem. Soc..

[183] Gates K.S. (2009). An overview of chemical processes that damage cellular DNA: Spontaneous hydrolysis, alkylation, and reactions with radicals. Chem. Res. Toxicol..

[184] Jaspers N.G., Raams A., Kelner M.J., Ng J.M., Yamashita Y.M., Takeda S., McMorris T.C., Hoeijmakers J.H. (2002). Anti-tumour compounds illudin S and Irofulven induce DNA lesions ignored by global repair and exclusively processed by transcription- and replication-coupled repair pathways. DNA Repair.

[185] Koeppel F., Poindessous V., Lazar V., Raymond E., Sarasin A., Larsen A.K. (2004). Irofulven cytotoxicity depends on transcription-coupled nucleotide excision repair and is correlated with XPG expression in solid tumor cells. Clin. Cancer Res..

[186] Otto C., Spivak G., Aloisi C.M., Menigatti M., Naegeli H., Hanawalt P.C., Tanasova M., Sturla S.J. (2017). Modulation of Cytotoxicity by Transcription-Coupled Nucleotide Excision Repair Is Independent of the Requirement for Bioactivation of Acylfulvene. Chem. Res. Toxicol..

[187] Casimir L., Zimmer S., Racine-Brassard F., Jacques P.E., Marechal A. (2023). The mutational impact of Illudin S on human cells. DNA Repair.

[188] Malvezzi S., Farnung L., Aloisi C.M.N., Angelov T., Cramer P., Sturla S.J. (2017). Mechanism of RNA polymerase II stalling by DNA alkylation. Proc. Natl. Acad. Sci. USA.

[189] Pommier Y., Kohlhagen G., Bailly C., Waring M., Mazumder A., Kohn K.W. (1996). DNA sequence- and structure-selective alkylation of guanine N2 in the DNA minor groove by ecteinascidin 743, a potent antitumor compound from the Caribbean tunicate Ecteinascidia turbinata. Biochemistry.

[190] Takebayashi Y., Pourquier P., Zimonjic D.B., Nakayama K., Emmert S., Ueda T., Urasaki Y., Kanzaki A., Akiyama S.I., Popescu N. (2001). Antiproliferative activity of ecteinascidin 743 is dependent upon transcription-coupled nucleotide-excision repair. Nat. Med..

[191] Son K., Takhaveev V., Mor V., Yu H., Dillier E., Zilio N., Pullen N.J.L., Ivanov D., Ulrich H.D., Sturla S.J. (2024). Trabectedin derails transcription-coupled nucleotide excision repair to induce DNA breaks in highly transcribed genes. Nat. Commun..

[192] Zewail-Foote M., Hurley L.H. (1999). Ecteinascidin 743: A minor groove alkylator that bends DNA toward the major groove. J. Med. Chem..

[193] Bueren-Calabuig J.A., Giraudon C., Galmarini C.M., Egly J.M., Gago F. (2011). Temperature-induced melting of double-stranded DNA in the absence and presence of covalently bonded antitumour drugs: Insight from molecular dynamics simulations. Nucleic Acids Res..

[194] Feuerhahn S., Giraudon C., Martinez-Diez M., Bueren-Calabuig J.A., Galmarini C.M., Gago F., Egly J.M. (2011). XPF-dependent DNA breaks and RNA polymerase II arrest induced by antitumor DNA interstrand crosslinking-mimetic alkaloids. Chem. Biol..

[195] Brooks P.J., Zakhari S. (2014). Acetaldehyde and the genome: Beyond nuclear DNA adducts and carcinogenesis. Environ. Mol. Mutagen..

[196] Xu L., Wang W., Wu J., Shin J.H., Wang P., Unarta I.C., Chong J., Wang Y., Wang D. (2017). Mechanism of DNA alkylation-induced transcriptional stalling, lesion bypass, and mutagenesis. Proc. Natl. Acad. Sci. USA.

[197] Voulgaridou G.P., Anestopoulos I., Franco R., Panayiotidis M.I., Pappa A. (2011). DNA damage induced by endogenous aldehydes: Current state of knowledge. Mutat. Res..

[198] Brooks P.J. (2007). The case for 8,5’-cyclopurine-2’-deoxynucleosides as endogenous DNA lesions that cause neurodegeneration in xeroderma pigmentosum. Neuroscience.

[199] Chatgilialoglu C., Ferreri C., Krokidis M.G., Masi A., Terzidis M.A. (2021). On the relevance of hydroxyl radical to purine DNA damage. Free Radic. Res..

[200] Brooks P.J. (2017). The cyclopurine deoxynucleosides: DNA repair, biological effects, mechanistic insights, and unanswered questions. Free Radic. Biol. Med..

[201] Kuraoka I., Bender C., Romieu A., Cadet J., Wood R.D., Lindahl T. (2000). Removal of oxygen free-radical-induced 5’,8-purine cyclodeoxynucleosides from DNA by the nucleotide excision-repair pathway in human cells. Proc. Natl. Acad. Sci. USA.

[202] Kropachev K., Ding S., Terzidis M.A., Masi A., Liu Z., Cai Y., Kolbanovskiy M., Chatgilialoglu C., Broyde S., Geacintov N.E. (2014). Structural basis for the recognition of diastereomeric 5’,8-cyclo-2’-deoxypurine lesions by the human nucleotide excision repair system. Nucleic Acids Res..

[203] Brooks P.J., Wise D.S., Berry D.A., Kosmoski J.V., Smerdon M.J., Somers R.L., Mackie H., Spoonde A.Y., Ackerman E.J., Coleman K. (2000). The oxidative DNA lesion 8,5’-(S)-cyclo-2’-deoxyadenosine is repaired by the nucleotide excision repair pathway and blocks gene expression in mammalian cells. J. Biol. Chem..

[204] Kirkali G., de Souza-Pinto N.C., Jaruga P., Bohr V.A., Dizdaroglu M. (2009). Accumulation of (5’S)-8,5’-cyclo-2’-deoxyadenosine in organs of Cockayne syndrome complementation group B gene knockout mice. DNA Repair.

[205] Wang J., Clauson C.L., Robbins P.D., Niedernhofer L.J., Wang Y. (2012). The oxidative DNA lesions 8,5’-cyclopurines accumulate with aging in a tissue-specific manner. Aging Cell.

[206] Krokidis M.G., D’Errico M., Pascucci B., Parlanti E., Masi A., Ferreri C., Chatgilialoglu C. (2020). Oxygen-Dependent Accumulation of Purine DNA Lesions in Cockayne Syndrome Cells. Cells.

[207] Cadet J., Davies K.J.A., Medeiros M.H., Di Mascio P., Wagner J.R. (2017). Formation and repair of oxidatively generated damage in cellular DNA. Free Radic. Biol. Med..

[208] Reardon J.T., Bessho T., Kung H.C., Bolton P.H., Sancar A. (1997). In vitro repair of oxidative DNA damage by human nucleotide excision repair system: Possible explanation for neurodegeneration in xeroderma pigmentosum patients. Proc. Natl. Acad. Sci. USA.

[209] Tornaletti S., Maeda L.S., Lloyd D.R., Reines D., Hanawalt P.C. (2001). Effect of thymine glycol on transcription elongation by T7 RNA polymerase and mammalian RNA polymerase II. J. Biol. Chem..

[210] Kitsera N., Stathis D., Luhnsdorf B., Muller H., Carell T., Epe B., Khobta A. (2011). 8-Oxo-7,8-dihydroguanine in DNA does not constitute a barrier to transcription, but is converted into transcription-blocking damage by OGG1. Nucleic Acids Res..

[211] Luhnsdorf B., Epe B., Khobta A. (2014). Excision of uracil from transcribed DNA negatively affects gene expression. J. Biol. Chem..

[212] Kitsera N., Allgayer J., Parsa E., Geier N., Rossa M., Carell T., Khobta A. (2017). Functional impacts of 5-hydroxymethylcytosine, 5-formylcytosine, and 5-carboxycytosine at a single hemi-modified CpG dinucleotide in a gene promoter. Nucleic Acids Res..

[213] Tornaletti S., Maeda L.S., Kolodner R.D., Hanawalt P.C. (2004). Effect of 8-oxoguanine on transcription elongation by T7 RNA polymerase and mammalian RNA polymerase II. DNA Repair.

[214] Kuraoka I., Suzuki K., Ito S., Hayashida M., Kwei J.S., Ikegami T., Handa H., Nakabeppu Y., Tanaka K. (2007). RNA polymerase II bypasses 8-oxoguanine in the presence of transcription elongation factor TFIIS. DNA Repair.

[215] Allgayer J., Kitsera N., von der Lippen C., Epe B., Khobta A. (2013). Modulation of base excision repair of 8-oxoguanine by the nucleotide sequence. Nucleic Acids Res..

[216] Allgayer J., Kitsera N., Bartelt S., Epe B., Khobta A. (2016). Widespread transcriptional gene inactivation initiated by a repair intermediate of 8-oxoguanine. Nucleic Acids Res..

[217] Fleming A.M., Burrows C.J. (2017). Formation and processing of DNA damage substrates for the hNEIL enzymes. Free Radic. Biol. Med..

[218] Shafirovich V., Kropachev K., Anderson T., Liu Z., Kolbanovskiy M., Martin B.D., Sugden K., Shim Y., Chen X., Min J.H. (2016). Base and Nucleotide Excision Repair of Oxidatively Generated Guanine Lesions in DNA. J. Biol. Chem..

[219] Shafirovich V., Kropachev K., Kolbanovskiy M., Geacintov N.E. (2019). Excision of Oxidatively Generated Guanine Lesions by Competing Base and Nucleotide Excision Repair Mechanisms in Human Cells. Chem. Res. Toxicol..

[220] Fung H., Demple B. (2005). A vital role for Ape1/Ref1 protein in repairing spontaneous DNA damage in human cells. Mol. Cell.

[221] Huang J.C., Hsu D.S., Kazantsev A., Sancar A. (1994). Substrate spectrum of human excinuclease: Repair of abasic sites, methylated bases, mismatches, and bulky adducts. Proc. Natl. Acad. Sci. USA.

[222] Tornaletti S., Maeda L.S., Hanawalt P.C. (2006). Transcription arrest at an abasic site in the transcribed strand of template DNA. Chem. Res. Toxicol..

[223] Wang W., Walmacq C., Chong J., Kashlev M., Wang D. (2018). Structural basis of transcriptional stalling and bypass of abasic DNA lesion by RNA polymerase II. Proc. Natl. Acad. Sci. USA.

[224] Okuda M., Suwa T., Suzuki H., Yamaguchi Y., Nishimura Y. (2022). Three human RNA polymerases interact with TFIIH via a common RPB6 subunit. Nucleic Acids Res..

[225] Carnie C.J., Acampora A.C., Bader A.S., Erdenebat C., Zhao S., Bitensky E., van den Heuvel D., Parnas A., Gupta V., D’Alessandro G. (2024). Transcription-coupled repair of DNA-protein cross-links depends on CSA and CSB. Nat. Cell Biol..

[226] Oka Y., Nakazawa Y., Shimada M., Ogi T. (2024). Endogenous aldehyde-induced DNA-protein crosslinks are resolved by transcription-coupled repair. Nat. Cell Biol..

[227] van Sluis M., Yu Q., van der Woude M., Gonzalo-Hansen C., Dealy S.C., Janssens R.C., Somsen H.B., Ramadhin A.R., Dekkers D.H.W., Wienecke H.L. (2024). Transcription-coupled DNA-protein crosslink repair by CSB and CRL4(CSA)-mediated degradation. Nat. Cell Biol..

